# Structure based analysis of K_ATP_ channel with a DEND syndrome mutation in murine skeletal muscle

**DOI:** 10.1038/s41598-021-86121-5

**Published:** 2021-03-23

**Authors:** Shoichiro Horita, Tomoyuki Ono, Saul Gonzalez-Resines, Yuko Ono, Megumi Yamachi, Songji Zhao, Carmen Domene, Yuko Maejima, Kenju Shimomura

**Affiliations:** 1 Department of Bioregulation and Pharmacological Medicine, Fukushima Medical University School of Medicine, 1 Hikarigaoka, Fukushima, 960-1295 Japan; 2 Department of Chemistry, University of Bath, Claverton Down, Bath, BA2 7AY UK; 3 Advanced Clinical Research Center, Fukushima Global Medical Science Center, Fukushima Medical University, Fukushima, Japan; 4 Chemistry Research Laboratory, University of Oxford, Mansfield Road, Oxford, OX1 3TA UK

**Keywords:** Cryoelectron microscopy, Computational biology and bioinformatics, Structural biology, Psychology

## Abstract

Developmental delay, epilepsy, and neonatal diabetes (DEND) syndrome, the most severe end of neonatal diabetes mellitus, is caused by mutation in the ATP-sensitive potassium (K_ATP_) channel. In addition to diabetes, DEND patients present muscle weakness as one of the symptoms, and although the muscle weakness is considered to originate in the brain, the pathological effects of mutated K_ATP_ channels in skeletal muscle remain elusive. Here, we describe the local effects of the K_ATP_ channel on muscle by expressing the mutation present in the K_ATP_ channels of the DEND syndrome in the murine skeletal muscle cell line C2C12 in combination with computer simulation. The present study revealed that the DEND mutation can lead to a hyperpolarized state of the muscle cell membrane, and molecular dynamics simulations based on a recently reported high-resolution structure provide an explanation as to why the mutation reduces ATP sensitivity and reveal the changes in the local interactions between ATP molecules and the channel.

## Introduction

Neonatal diabetes (ND) is characterized by the development of hyperglycemia within the first 6 months of life resulting from impaired insulin secretion caused by gain-of-function mutations in the ATP-sensitive potassium (K_ATP_) channel^1–11^. The most severe phenotype of ND is known as the DEND (the developmental delay, epilepsy, and neonatal diabetes) syndrome which patients experience developmental delay, epilepsy, muscle weakness and hyperglycemia^10–18^. The muscle weakness observed in DEND syndrome patients is considered to originate in the brain^19^. However, because the K_ATP_ channel is also expressed in skeletal muscle, a mutated K_ATP_ channel may contribute to the development of muscle symptoms in DEND syndrome patients.

The K_ATP_ channel plays pivotal roles in the regulation of insulin secretion^20–24^. When glucose metabolism is enhanced in response to high glucose concentrations in pancreatic beta-cells, metabolically-generated ATP closes the K_ATP_ channels, thus causing membrane depolarization and action potential firing, leading to insulin secretion^25^. The K_ATP_ channel is composed of two subunits: a Kir6.x (Kir6.1 and Kir6.2) channel and a sulfonylurea receptor (SUR; SUR1, SUR2A and SUR2B)^26–30^. Pancreatic beta-cells and neurons are known to mainly express in a combination of Kir6.2/SUR1 and the K_ATP_ channels in skeletal muscle are composed of Kir6.2/SUR2A^31,32^. The tetrameric Kir6.2 is a K^+^-channel and together with the channel regulating SUR subunits, they form a heteromeric complex in the cell membranes^26–28^. The interface between Kir6.2 subunits forms an ATP binding pocket, and ATP recruited from the cytosol drives structural rearrangement that results in the closure of the channel. Therefore, mutated Kir6.2 channels may have direct effect on muscle function in DEND syndrome patients. Moreover, the physiological role of the sarcoK_ATP_ channel remains unclear^33,34^. In the present study, we constructed an adeno-associated viral (AAV) vector and transfected the mouse myoblast cell line C2C12 with mutation in the Kir6.2 subunit (Kir6.2-R50P) which results in DEND syndrome^17^, and we investigated its effects on cell differentiation, myogenic index, myotube width, membrane potential and glucose uptake activity. In combination with molecular dynamics simulations of the wild type as well as the single mutant R50P, based on the most recently resolved structure^35–40^, we discuss the structural implications in the function and physiological role of the sarcoK_ATP_ channel.

## Results

### Hyperpolarized state in Kir6.2 (R50P)-expressing myotube

**Figure 1:**
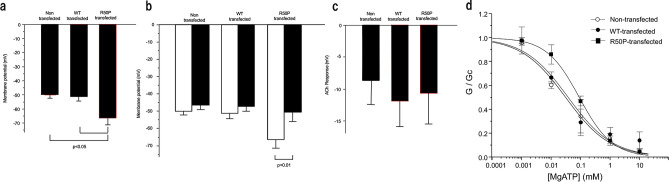
Membrane potential and MgATP sensitivity of Kir6.2/R50P myotubes. (**a**–**c**) Mean membrane potential (± SEM) of C2C12 myotube (**a**), before (white) and after (black) application of glibenclamide (**b**), and ACh response (**c**) without infection (N = 5–6), with infection of the Kir6.2/WT AAV vector (N = 5–6), the Kir6.2/R50P AAV vector (N = 5–6). Multiple groups were compared using an ordinary One-Way ANOVA test, followed by Tukey’s test, and two groups were compared using Paired t test. (**d**) Mean relationship between MgATP and K_ATP_ channel conductance (*G*), expressed as relative to the conductance in the absence of nucleotide (*Gc*) for non-transfected (n = 4: open circle), WT transfected (n = 4: filled circle) and Kir6.2-R50P transfected (n = 4: closed square). The smooth curves are best fit of the equation shown in methods section to the mean data. For non-transfected cells, IC_50_ = 0.038 mM, h = 0.58. For WT-transfected cells, IC_50_ = 0.031 mM, h = 0.58. For Kir6.2-R50P transfected cells, IC_50_ = 0.09 mM, h = 0.78. The data were analyzed and drawn with Origin Graphing and Data Analysis Software (Version 9; https://www.originlab.com).

**Figure 2:**
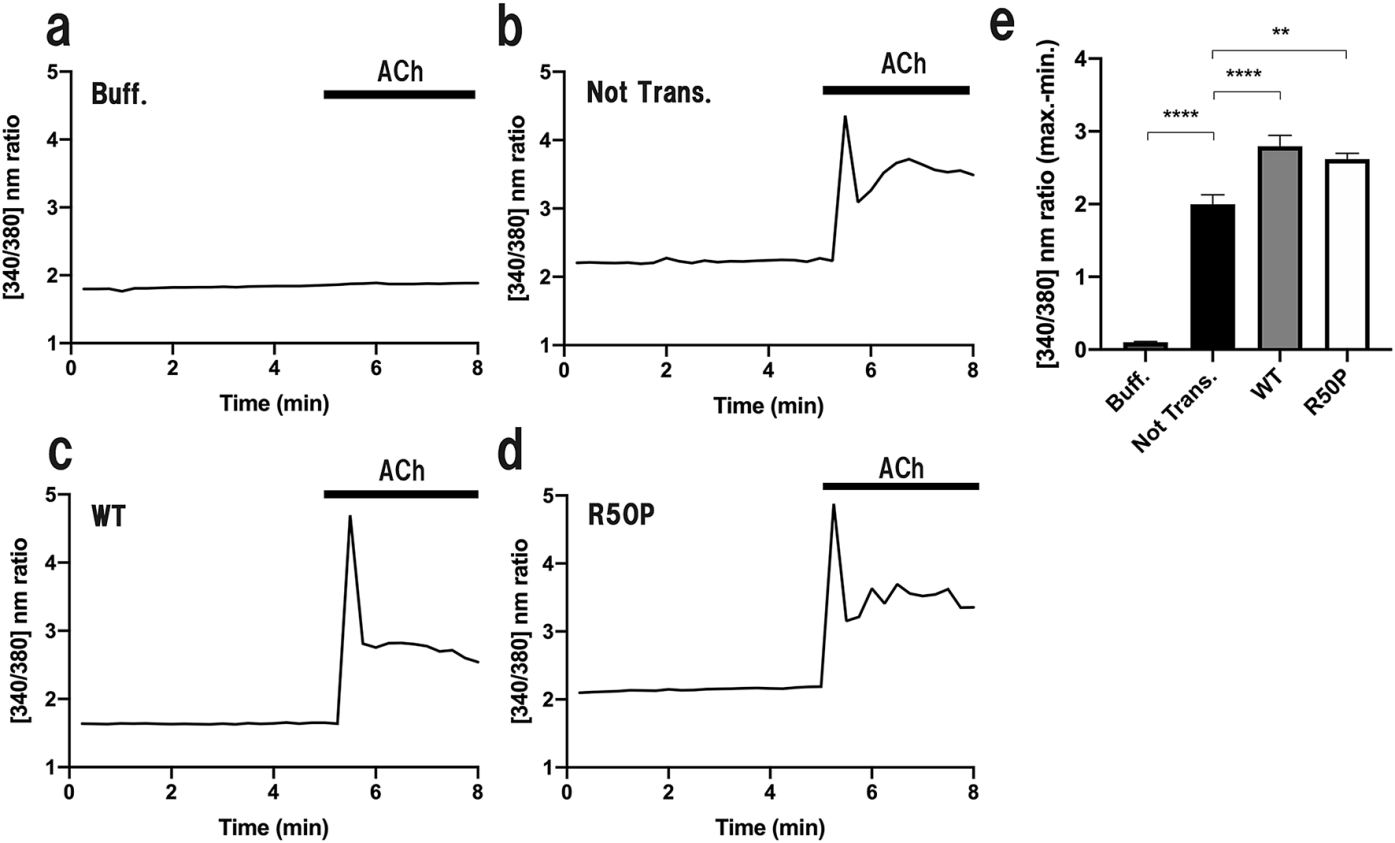
Cytosolic Ca^2+^ release in Kir6.2/R50P-expressing myotubes. (**a**–**d**) Representative changes of cytosolic Ca^2+^ ([Ca^2+^]_i_) images applying buffer (**a**) and 10 mM ACh (**b**–**d**) to non-transfected (**b**), Kir6.2/WT-expressing (**c**) and Kir6.2/R50P-expressing myotubes (**d**). (**e**) Mean firing frequency (± SEM) before and after 10 mM ACh stimulation at 5 min (N = 6–12). Multiple groups were compared by an ordinary one-way ANOVA test, followed by Tukey’s test (**p < 0.01; ****p < 0.001). The figures were analyzed and created with GraphPad Prism software (GraphPad Prism 8.2.1; https://www.graphpad.com/scientific-software/prism/).

The exit from the endoplasmic reticulum to the plasma surface acts as a checkpoint for controlling both Kir6.2 and SUR subunits, and a three-amino acid trafficking sequence (RKR) was found to prevent surface expression of the Kir6.2 and SUR subunits^41^. Co-assembly of both subunits shows profound enhancement of plasma surface expression compared with each subunit expressed alone^24,42^. Therefore, without the expression of the SUR regulatory subunit, Kir6.2 is not expressed in the plasma membrane, and almost no channel activity is exhibited. We examined the effect of the Kir6.2/R50P mutation on C2C12 cells by transfecting wild-type Kir6.2 (Kir6.2/WT) or DEND inducing Kir6.2 (Kir6.2/R50P). Heterozygous expression of Kir6.2 (Kir6.2/R50P) is supposed to increase the population of Kir6.2 (Kir6.2/R50P)/SUR on the plasma membrane surface. The cells we employed should reflect the condition of the patients because DEND syndrome patients are heterozygous with a mutated K_ATP_ channel. C2C12 cells express endogenous SUR, and therefore Kir6.2 transfected cells should express functioning K_ATP_ channels in combination with Kir6.2 and SUR. The expression of Kir6.2 were examined by quantitative reverse transcription polymerase chain reaction (qRT-PCR) and immunohistochemical analyses (Table S1, Figs. S1, S2). As expected, the endogenous Kir6.2 were confirmed in both non-transfected and transfected myotubes whereas virally expressed Kir6.2 were confirmed in transfected myotubes only. Also, it was found that the C2C12 myotubes express SUR1 in addition to SUR2A. Furthermore, we have also confirmed the differentiation by increased mRNA expression levels of Kir6.2 (3.3-fold) and SUR2A (15.8-fold) (Fig. S3). We applied current clamp mode in standard-whole cell technique to measure the membrane potentials which were consistent to the previous study^43^. Electrophysiological analyses demonstrated that the membrane potential difference between non-transfected and Kir6.2/WT-expressing myotubes exhibit resting potentials of similar order of magnitude (Fig. 1a): − 50.0 ± 2.3 mV for non-transfected cells and − 51.3 ± 3.2 mV for Kir6.2/WT expressing cells. However, the difference between non-transfected and the Kir6.2/R50P-expressing myotubes exhibit significantly a more hyperpolarized state with values of − 66.6 ± 4.9 mV for Kir6.2/R50P expressing cells: p < 0.05. Similar results were obtained when using myoblasts (Fig. S4); the membrane potential between non-transfected and Kir6.2/WT-expressing myoblasts was similar (− 14.7 ± 2.0 mV for non-transfected cells and − 16.0 ± 4.8 mV for Kir6.2/WT expressing cells). However, the Kir6.2/R50P-expressing myoblasts were significantly more hyperpolarized than the non-transfected myoblasts (− 14.7 ± 2.0 mV for non-transfected cells and − 25.3 ± 2.9 mV for Kir6.2/R50P expressing cells: p < 0.05). The results indicate that the wild-type K_ATP_ channel in skeletal muscles has little impact on the resting membrane potential but R50P mutation can shift membrane potential to hyperpolarization. To confirm that this hyperpolarizing shift of R50P-expressing cells is indeed induced by the mutated K_ATP_ channel, we applied K_ATP_ channel blocker, 100 μM glibenclamide, and measured the change of membrane potential. As shown in Fig. 1b, glibenclamide had no effect on membrane potentials of non-transfected cells (− 50.0 ± 2.3 mV before, − 46.5 ± 2.6 mV after application of glibenclamide) and WT-transfected cells (− 51.3 ± 3.2 mV before, − 47.3 ± 2.8 mV after application of glibenclamide). However, the application of glibenclamide increased the membrane potential of R50P-transfected cells to similar level as non-transfected and WT-transfected cells (− 66.6 ± 4.9 mV before, − 50.5 ± 5.5 mV after application of glibenclamide). These results indicate that R50P mutation is responsible for the hyperpolarized shift of membrane potential observed in the present study. Also, the MgATP dose–response curves show that Kir6.2/R50P-expressing myotubes have less ATP sensitivity compared to Kir6.2/WT-expressing or non-transfected myotubes (Fig. 1d). Taken together, the R50P mutation induces a reduction of the membrane potential within the physiological range of intracellular MgATP concentrations, and this in turn may result in a reduction of the acetylcholine (ACh) stimuli. Therefore, we subsequently examined whether depolarization induced by 10 mM acetylcholine (ACh) differ in Kir6.2/R50P-expressing myotubes (Fig. 1c). We concluded that there were no significant differences when examining on the cell line basis in response to ACh (− 12.2 ± 2.1 mV for non-transfected cells; − 12.2 ± 3.8 mV for Kir6.2/WT-expressing cells; − 10.9 ± 4.8 mV for Kir6.2/R50P-expressing cells). In addition, the results of the Ca^2+^ release from the intracellular Ca^2+^ store in the endoplasmic reticulum in Kir6.2/R50P-expressing myotubes showed that there is not a significant difference between Kir6.2/WT- and Kir6.2/R50P-expressing myotubes in response to the same concentration of ACh (Fig. 2). As downstream factor, the qRT-PCR of Baf60c, Deptor and AKT2 revealed that no significant difference was found in these factors in our cell line system (Fig. S1).

### Structural analyses and molecular dynamics simulations of R50P polymorphism in Kir6.2

**Figure 3:**
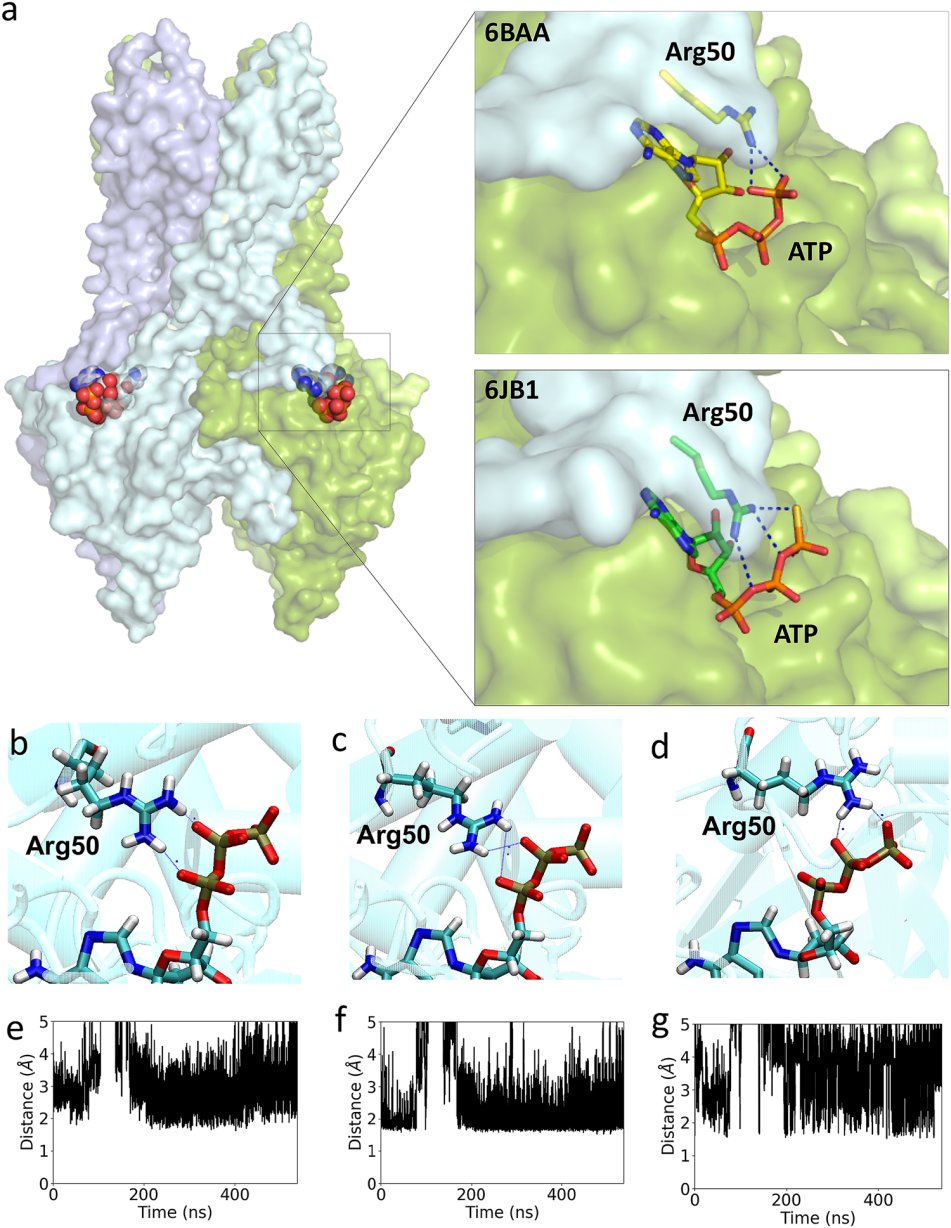
Arg50 plays pivotal roles in ATP binding from structural analyses. (**a**) The overall structure of the Kir6.2 tetramer extracted from the K_ATP_ channel (6BAA, 6JB1) drawn with PyMol (http://pymol.sourceforge.net/), both of which are solved with the highest resolution reported thus far (6BAA, 3.6 Å; 6JB1, 3.3 Å). The structure clearly reveals that the Arg50 residue is located at the loop region for twining around the subsequent Kir6.2 subunit. The side chain of Arg50 electrostatically interacts with the gamma phosphate group of ATP in 6BAA, whereas the residue extensively interacts with beta and gamma phosphate groups in ATP. (**b**–**d**) Representative interactions between Arg50 and alpha/beta/gamma phosphate groups in ATP drawn with VMD^55^. (**e**–**g**) The distance between Arg50 HH12–ATP O3A (**e**), Arg50 HH12–ATP O3B (**f**), Arg50 HH12–ATP O3G (**g**) were measured for 500 ns molecular dynamics simulation. All plots were produced with xmgrace (http://plasma-gate.weizmann.ac.il/Grace/). Analyses for other monomers and replicas are presented in the Supplementary Materials.

The R50P-mutated K_ATP_ channel exhibits gain-of-function properties with reduced ATP sensitivity^17,18^. Structural analyses from recently resolved structures^37,40^ (PDB IDs: 6BAA and 6JB1) show that the Arg50 residue is located at a solvent exposed loop in the intracellular region of the Kir6.2 subunit, and that this residue facilitates wrapping around the nearby Kir6.2 subunit (Fig. 3a). Due to the spatial proximity, the side chain of Arg50 establishes electrostatic interactions with the phosphate groups in ATP, facilitating ATP sensing in the cytosol. To further characterize the ATP binding site and the interactions between ATP and the channel residues, molecular dynamics simulations were performed on Kir6.2/WT and Kir6.2/R50P inserted in a model membrane of POPC (1-palmitoyl-2-oleoyl-sn-glycero-3-phosphocholine).

A simple measure of the stability of the structure during the dynamics is given by the drift from its initial conformation provided by the root-mean-square deviation (RMSD) between the channel structure at a given time and the initial structure. Analysis of the alpha carbon atom RMSD for the entire tetramer, and the M1 (residues 67–97) and M2 (residues 142–172) helices versus time revealed a similar pattern in each of the simulations (Fig. S5). The initial structural drift reflects the fact that the restraints imposed during the equilibration period have been switched off and that the protein is free to relax within the lipid bilayer and the solvent. The overall RMSD values are the same for all the simulations suggesting overall conformational stability of the protein at these timescales.

While Martin et al*.* reported that Arg50 interacts with only the gamma phosphate group (PDB ID: 6BAA; 3.6 Å-resolution)^37^, Chen et al.reported that the residue extensively interacts with the beta and gamma phosphate groups (PDB ID: 6JB1; 3.3 Å-resolution)^40^ (Fig. 3a). In line with Chen’s reports, the hydrogen atoms of the sidechain of Arg50 interact with the beta and gamma phosphate groups of ATP in our simulations of the wild type (Fig. 3d,f,g, Fig. S6). In addition, interactions with the alpha phosphate group of ATP are also recorded (Fig. 3b,c,e). Replacement of Arg50 with a Pro residue results in the loss of the extensive electrostatic interactions established between Arg50 and the ATP molecules. Furthermore, the relatively rigid five-membered ring of the Pro replacement drastically limits the range of the values of the torsion angle φ (rotation about N–C^α^ bond of the peptide backbone) to approximately − 60°, leading to loss of flexibility.

**Figure 4:**
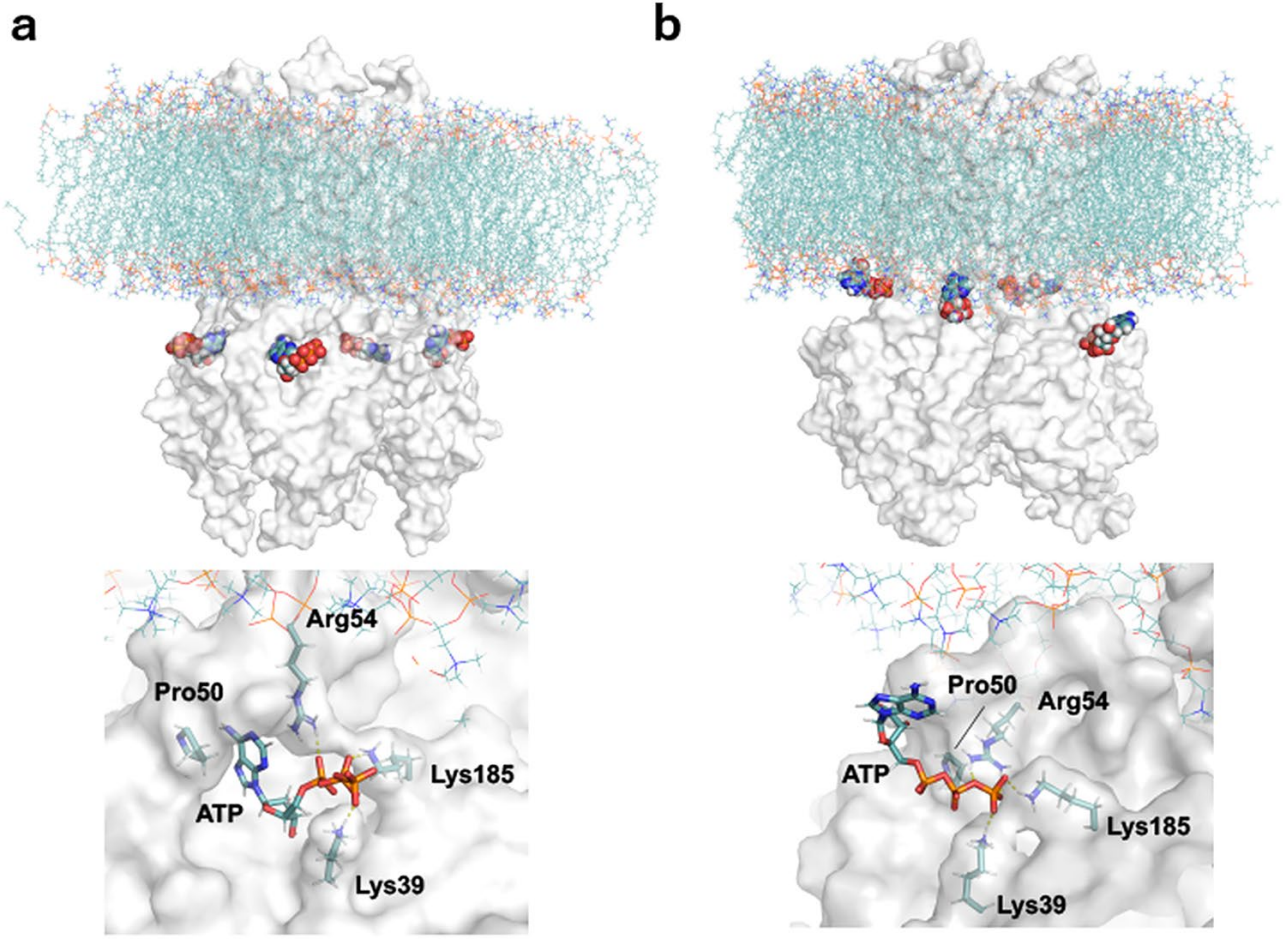
Comparison between before and after molecular dynamics simulation in R50P mutant. Before simulation, four ATP molecules were accommodated in the cavity (**a**), whereas after 500 ns, each adenosine moiety in the ATP is exposed to the solvent (**b**). The ATP molecule is weakly bound to the channel with three residues Lys39, Arg54, Lys185. The simulation was calculated by gromacs v2020.1 software (https://doi.org/10.5281/zenodo.3562512) and the figure was drawn in PyMol (http://pymol.sourceforge.net/).

During the simulations, interactions between ATP and nearby residues were recorded as a function of time. It was found that residues Lys185, Lys39 and Arg54 as well as Arg50 in the wild-type and Gln52 in the mutant are in close contact with the ATP molecules at the beginning of the simulations. Some of these interactions are maintained during the entire simulation time and in some other instances, there is an exchanged between the pair of atoms of a given residue that interact with the ATP molecule (Fig. 3b–d). While four ATP molecules remain bound to the wild-type Kir6.2 through their robust links with residues Lys185 and Arg50, and to some extend Arg54 and Lys39 in the absence of SUR1 subunits, the majority of the ATP-protein interactions diminishes as the simulation progresses in the case of the R50P mutant simulations (Table 1). Overall, the association between ATP and the protein is weaker in the R50P mutant compared to the wild type as the interactions described between the oxygen atoms from the phosphate groups of ATP and the hydrogen atoms of the guanidinium side chain of arginine are impaired (Fig. 4, Fig. S6). During the simulations, the side chain of Lys39 interacts with the phosphate groups of ATP, facilitating the association of ATP with the protein although the final orientation of ATP is far from its original state. Notably, residues Arg50 and Arg54 of the same monomer are in direct contact with a SUR subunit and likewise, residues Lys39 and Lys185 each in a nearby monomer also interact directly with the same SUR subunit. Only the interactions between Lys185 and the ATP molecules remain in place in both the wild type and the mutant in the four monomers of the protein during the whole time in all the replicas computed, although there are signs of weakening as the time progresses. The simulation results agree with the observation that the mutation K185Q is also involved in the DEND syndrome^44^. Although the simulations were carried out in the absence of the SUR subunits, altering these SUR-Kir interactions could potentially affect the function of the complex.

**Table 1:** Summary of simulations performed and the interactions established between ATP molecules and the Kir6.2 channel residues after 500 ns of simulation time in the wild type and mutant systems considered. Arg50 is used for ATP binding in all four monomers in wild type.

System	Replica Number	Protein-ATP interactions (presence in # monomers)
Wild type	1	Lys185 (4)/Arg54 (2)/Arg50 (4)/Lys39 (4)
	2	Lys185 (4)/Arg54 (2)/Arg50 (4)/Lys39 (4)
	3	Lys185 (4)/Arg54 (2)/Arg50 (4)/Lys39 (2)
	4	Lys185 (4)/Arg54 (2)/Arg50 (4)/Lys39 (4)
R50P	1	Lys185 (4)/Arg54 (3)/Gln52 (1)/Lys39 (3)
	2	Lys185 (4)/Arg54 (3)/Gln52 (2)/Lys39 (3)
	3	Lys185 (4)/Arg54 (2)/Gln52 (0)/Lys39 (3)
	4	Lys185 (4)/Arg54 (2)/Gln52 (1)/Lys39 (1)

### Effect of myogenesis and glucose uptake in Kir6.2/R50P-expressing myotubes

**Figure 5:**
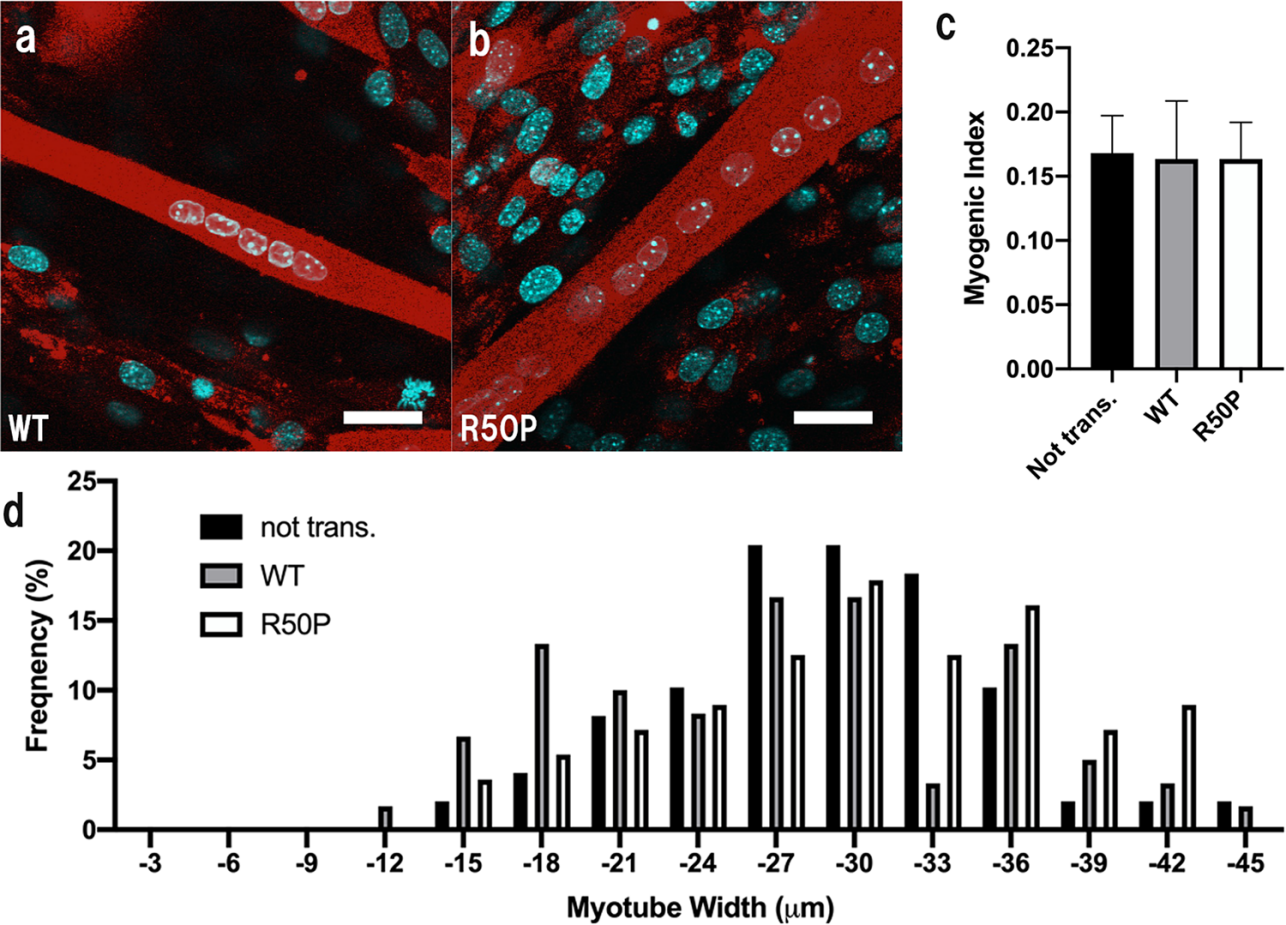
Effects of R50P mutation to skeletal muscle myogenesis. (**a**,**b**) Kir6.2/WT and Kir6.2/R50P expression in a C2C12 myotube cultured for 96 h after AAV vector infection coding (**a**) Kir6.2/WT (**b**) Kir6.2/R50P. Representative images are shown. Scale bar 20 μm. (**c**,**d**) R50P mutation does not affect the myogenic index as described in the text. Data are the mean ± SEM per treatment group, each examining 20 randomly selected fields. Mean myotube widths (**c**) and distribution of myotube widths (**d**) were calculated for each treatment group. The figures were created by GraphPad Prism software (GraphPad Prism 8.2.1).

**Figure 6:**
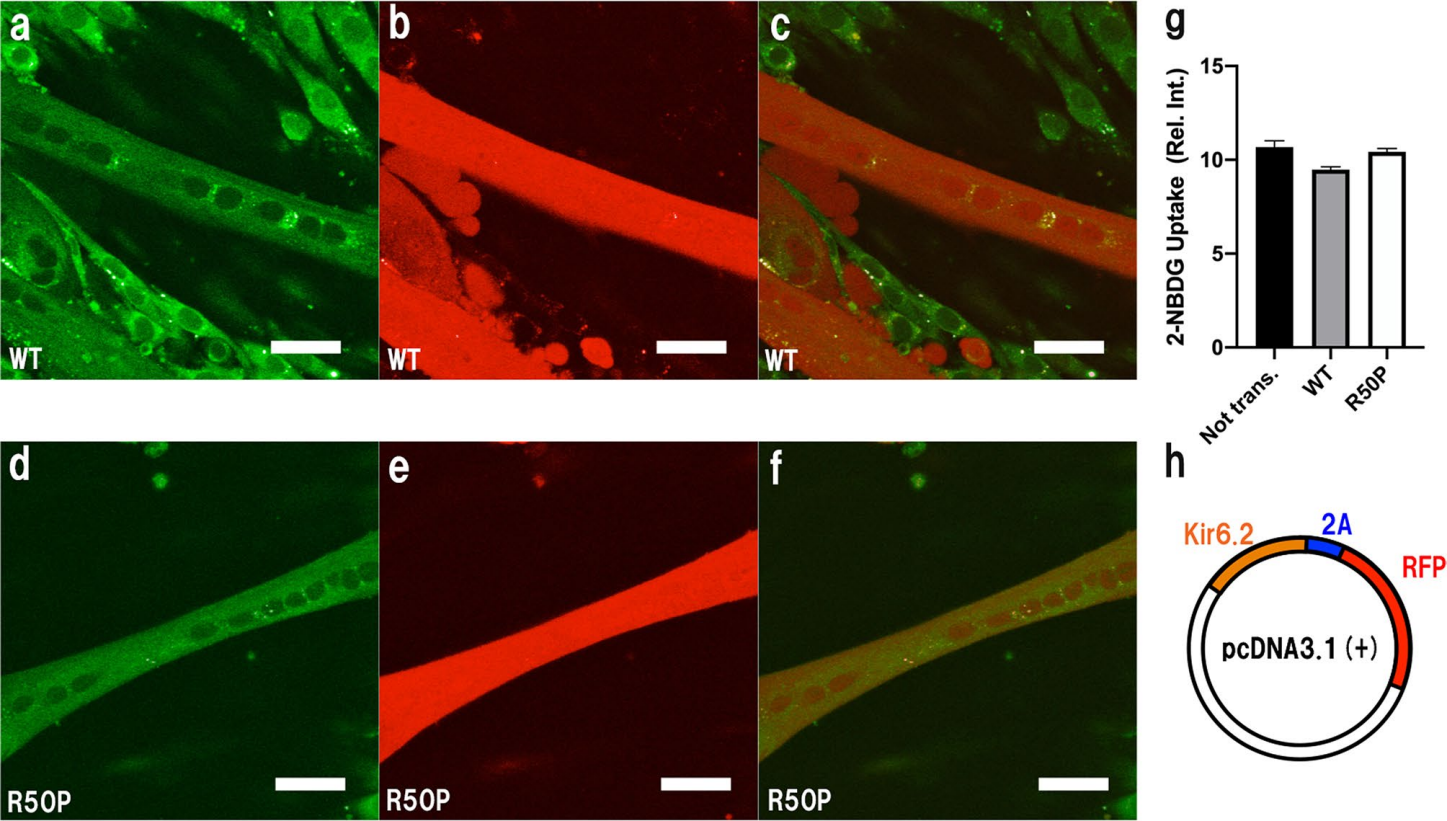
Effects of glucose uptake in Kir6.2/R50P-expressing myotubes. (**a**–**c**) Confocal laser-scanning microscope images of Kir6.2/WT-expressing myotubes. 2-NBDG fluorescence (**a**), Kir6.2/WT localization (**b**), merged image of (**a**,**b**) (**c**). (**d**–**f**) Confocal laser-scanning microscope images of Kir6.2/R50P-expressing myotubes. 2-NBDG fluorescence (**d**), Kir6.2/R50P localization (**e**), merged image of (**d**,**e**) (**f**). Scale bars 100 μm. (**g**) Mean value of 2-NBDG uptake between non-transfected, Kir6.2/WT, Kir6.2/R50P-expressing myotubes. (**h**) pAAV vector design, the Kir6.2-expresing myotubes show red fluorescence. The intensities were measured using ImageJ software (Version 1.51; https://imagej.nih.gov/ij/download.html), and the figures were analyzed and created by GraphPad Prism software (GraphPad Prism 8.2.1).

To examine the physiological effects of the R50P mutation of the K_ATP_ channel on skeletal muscle, myogenesis and glucose uptake measurements were performed. As shown in Fig. 5, the myogenic index values between non-transfected Kir6.2/WT- and Kir6.2/R50P-expressing murine skeletal muscles were 0.168 ± 0.029, 0.164 ± 0.045, 0.163 ± 0.029, respectively, which suggests that K_ATP_ channels are not involved in the myogenic index. At the same time, myotube width analyses show that both Kir6.2 (WT)-expressing and Kir6.2 (R50P)-expressing myotubes exhibit almost similar width distribution with a maximum peak at 27–30 μm. Taken together, these results indicate that K_ATP_ channels are not involved in myogenesis on a cell line basis. Subsequently, glucose uptake was evaluated with confocal laser-scanning microscopy. The mean average values of the green fluorescence were recorded for the glucose uptake in Kir6.2/WT- and Kir6.2/R50P-expressing murine skeletal myotubes. Figure 6 shows that, in the presence of insulin, the mean average value of the green fluorescence of the Kir6.2/R50P-expressing myotubes is almost similar to that of the Kir6.2/WT-expressing myotubes, indicating that K_ATP_ channels are not involved in glucose uptake on a cell line basis.

## Discussion

A previous study demonstrated that muscle dysfunction was of neuronal origin by comparing muscle- and nerve-specific expression of the Kir6.2/V59M mutation in transgenic mice (named m-V59M and n-V59M mice, respectively), the most commonly known mutation inducing intermediate DEND syndrome^19^. Interestingly, there was no significant difference in muscle membrane potential between wild-type and m-V59M mice or wild-type and n-V59M mice, indicating that the gain-of-function mutation in the K_ATP_ channel does not directly affect muscle function itself. However, the present study shows that the R50P mutation, DEND syndrome inducing mutation with more severe symptoms than the V59M, induces a hyperpolarized membrane potential. Although the muscle weakness is mainly originated in the brain, our present data indicate that severely reduced ATP sensitivity in the muscle K_ATP_ channels may take part to develop muscle weakness in DEND syndrome patients. From the recent K_ATP_ channel structures solved at atomic resolution, it is inferred that Arg50 can play pivotal roles in the recognition of nucleotides by establishing electrostatic interactions with ATP, and it is indeed part of the ATP binding pocket in Kir6.2^40^. It is well established that this mutation decreases ATP sensitivity without affecting the open probability of the channel (denoted “binding mutation”)^17^. Our present simulation results further confirmed that R50P mutation indeed contributes to reduce the ATP sensitivity. It is of interest because the simulation enabled us to see the serial interaction between ATP and its binding residues while released from the binding pocket (such as the K185Q mutation described above), which facilitates the understanding of molecular bases of ATP binding mechanism and development of neonatal diabetes/DEND syndrome. In contrast, Val59, which has been reported to affect ATP sensitivity with increased open probability (denoted “gating mutation”)^19^ is located at a distal place, > 18 Å away from the ATP location (PDB ID: 6JB1) and not contributing directly to ATP binding. Consequently, the effect on the activation mechanism of each of these two mutations seems to be rather different from an atomistic perspective.

Inside-out patch clamp experiments demonstrated that the C-terminal tail comprised by 42 a.a. residues in SUR2A possess an inhibitory effect on NBD2-mediated ADP-induced channel activation^45^. In line with these observations, higher concentrations of ADP were required to activate K_ATP_ channels that contained more SUR2A than SUR1 or SUR2B regulatory domains^46^. As a consequence, the K_ATP_ channel with the SUR2A subunit tended to be found in a closed state. Therefore, it is possible that a “binding mutation”, such as R50P, has higher impact on ATP sensitivity in SUR2A-containing K_ATP_ channels compared to “gating mutations” such as V59M, which showed no difference in membrane potential in skeletal muscle. As suggested by the recently reported K_ATP_ channel atomic structure^35–40^, SUR1 adopts an inward-facing conformation which is an inactive form, with a folded lasso motif that facilitates ATP binding in Kir6.2. In contrast, the lasso motif transforms to an extended form when the complex adopts an outward-facing structure. Considering these observations, given that SUR2A adopts an inward-facing conformation more likely than SUR1, the R50P mutation may disrupt SUR2A regulation more drastically than the SUR1 regulation, which is consistent with the previous observations^46^. Consequently, the R50P “binding mutation” in Kir6.2 may have a higher impact on the membrane potential than the “gating mutation” in skeletal muscles, or even in cardiomyocytes^47–50^.

In this study, we used C2C12 cells with transfected WT and R50P-mutated Kir6.2. Since Kir6.2 and SUR are both required to be expressed in cell surface, transfected Kir6.2 channel surface expression should be under the regulation of endogenous SUR. Because except for the rare case, most of the reported neonatal diabetes/DEND syndrome patients are heterozygous with mutated channel subunit^51^, we consider that our system is reflecting the heterozygous state of the patients without excessive expression of K_ATP_ channels in cell surface.

In previous study, ATP sensitivity experiments show that in pancreatic Kir6.2/R50P-SUR1 heteromeric K_ATP_ channel, approximately 33% channel current is unblockable^17^. However, high dose of MgATP was able to fully block K_ATP_ channel current in Kir6.2/R50P transfected C2C12 cells (Fig. 1d). Therefore, although we need to keep in mind that the current we have recorded in this study should reflect the mixture of Kir6.2/SUR1 and Kir6.2/SUR2A (Fig. S1), it can be considered that R50P mutation has less impact on skeletal muscle compared to that of pancreas and brain, and that the R50P mutated neuronal K_ATP_ channel may mainly contribute to muscle weakness. Nevertheless, this study shows that R50P mutated sarcoK_ATP_ channel does contribute to hyperpolarization unlike V59M mutated sarcoK_ATP_ channel. Furthermore, this study revealed that the application of glibenclamide shows no effects on non-transfected and Kir6.2 (WT)-expressing myotubes, whereas the application partly restores the hyperporalized state in Kir6.2 (R50P)-expressing myotubes. These results indicate that in skeletal muscle, wild-type K_ATP_ channels are closed in physiological state with very small contribution on membrane potential and also indicating the possible effects of glibenclamide on DEND syndrome patients. Also, we need to keep in mind that glibenclamide is reported to act on activate exchange protein directly activated by cAMP 2 (Epac2A) or carnitine palmitoyltransferase 1 (CPT-1)^52–55^. Epac2A is not expressed in skeletal muscle but CPT-1 is known to be expressed in skeletal muscle and functions as the key regulatory enzyme of mitochondrial long-chain fatty acid oxidation. Therefore, further study is required to clarify the targets of glibenclamide other than K_ATP_ channels and its function in skeletal muscle.

It is possible that the hyperpolarization caused by the R50P mutation may affect downstream targets of muscle cells, and Meng et al. has shown that Baf60c-Deptor-AKT2 axis is affected by the activities of K_ATP_ channel in skeletal muscle^56^. Therefore, we have investigated whether opening of K_ATP_ channel induced by R50P mutation has any effect on the downstream signaling of the channel. The Baf60c-Deptor-AKT2 axis was found not to be affected by the R50P mutation (Fig. S1). Furthermore, the intracellular Ca^2+^ levels and glucose uptake were also not affected by this mutation (Figs. 2, 6). This was of surprise since previous studies using Kir6.2 or SUR2 KO mice showed changes in intracellular Ca^2+^ or glucose uptake^57,58^.

As for the limitation, data from the transgenic mice with Kir6.2 (R50P) or any other neonatal diabetes inducing mutation are not available in this study. However, the present study provided insight on the links between muscle weakness and sarcoK_ATP_ channel. Further study using a transgenic model of the muscle-specific Kir6.2(R50P)-expressing mouse is required to clarify the mechanism of muscle weakness in the DEND syndrome patients.

## Materials

### Myogenic cell culture

The murine myoblasts and myotubes were prepared as described in the previous study^59^. Briefly, the murine C2C12 (muscle myoblast) cell line (code: CRL-1772) was purchased from the American Type Culture Collection (Manassas, VA, USA). Myoblasts were cultured at 37 °C in a humidified atmosphere of 5% CO_2_ in growth medium consisting of high-glucose Dulbecco’s modified Eagle’s medium (DMEM; Wako, Osaka, Japan), 10% (v/v) heat-inactivated fetal bovine serum (FBS) (Equitech Bio, Kerrville, TX, USA), 100 U/mL penicillin (Wako), and 100 μg/mL streptomycin (Wako). When the cells reached 70–80% confluence, the culture medium was changed to a differentiation medium to induce myogenesis. The differentiation medium consisted of high-glucose DMEM, 2% heat-inactivated horse serum (Thermo Fisher Scientific, Waltham, MA, USA), 100 U/mL penicillin (Wako), and 100 μg/mL streptomycin (Wako).

### Viral vector preparation

The plasmid pAAV vector, which was provided by K. Kobayashi, encoded the EF1α promoter, 2A self-cleaving peptide, red fluorescent protein (RFP) and woodchuck hepatitis virus posttranscriptional regulatory element (WPRE) sequences. The point mutant (R50P) of Kir6.2 was generated by using the wild-type Kir6.2 cDNA as templates and primers 5′-GGG AAC TGC AAC GTA GCC CAC AAG AAC ATA CCT GAG CAG GGT CGC TTC TTG CAG GAC GTG TTT-3′ and 5′-AAA CAC GTC CTG CAA GAA GCG ACC CTG CTC AGG TAT GTT CTT GTG GGC TAC GTT GCA GTT CCC-3′. Amplified fragments of Kir6.2 (WT) and Kir6.2 (R50P) were incorporated into the pAAV vectors described above. Homogeneous recombination was performed by using the in-fusion HD cloning kit (Takara Bio, Shiga, Japan). Around 50% of confluent HEK293T cells grew in high-glucose DMEM growth medium supplemented with 10%(v/v) FBS, 100 U/mL penicillin and 100 μg/mL streptomycin were triple transfected with a pHelper vector (11,635 bp, Takara Bio), a pRC2-mi342 vector (8189 bp, Takara Bio), and the above-described pAAV vector encoding the Kir6.2 (WT) or Kir6.2 (R50P) gene plasmids, using a HBSP solution (5 mM HEPES pH 7.1, 140 mM NaCl, 0.75 mM Na_2_HPO_4_, 5 mM KCl, 6 mM D( +)-Glucose). After transfection for 72 h, cells were harvested by centrifugation (1000*g*, 3 min), resuspended in TBS Buffer (25 mM Tris pH 7.4, 137 mM NaCl, 2.68 mM KCl) and disrupted by freeze–thaw cycles (three times). After disruption, benzonase (Novagen Millipore) was added and incubated with DNaseI for 30 min at 37 °C. After centrifugation (10,000*g*, 15 min), the centrifugation tubes with CsCl_2_ dissolved in phosphate-buffered saline (PBS) and the supernatant were centrifuged (55,000*g*) for 24 h at 16 °C. This CsC_l2_-based purification step was repeated twice with two CsCl_2_ concentrations (1.24 g/mL and 0.55 g/mL). After gravity flow purification, each fraction was confirmed by reverse transcription polymerase chain reaction (RT-PCR) using the WPRE sequence, and dialyzed three times to replace the Cs containing buffer with PBS buffer. After concentrated approximately to 100 μL, the virus was frozen at -80 °C. The genomic titer was determined by RT-PCR.

### Structural analyses and molecular dynamics simulations

The K_ATP_ channel structures were selected from the Protein Data Bank (https://www.rcsb.org), PDB IDs: 6C3O; 6C3P; 6BAA; 5WUA; 5TWV; 5YKE; 5YKF; 5YKG; 5YWA; 5YWB; 5YWC; 5YW8; 5YW9; 6JB1, all of which were obtained by cryogenic electron microscopy^35–40^. Structures with resolution higher than 4 Å were selected: 6C3O (3.9 Å), 6BAA (3.6 Å), 6JB1 (3.3 Å). The Arg50 residue in Kir6.2 is only assigned in PDBs 6BAA (3.6 Å) and 6JB1 (3.3 Å), and thus, these two structures were selected for structural analyses using PyMol (http://pymol.sourceforge.net/). To begin to address the structural mechanisms by which the mutations considered in this study alter the action of ATP on the Kir6.2 ^+^-channel, molecular dynamics simulations were carried out using the wild type channel and the mutant R50P. The crystal structure of Kir6.2 closed at its inner gate was retrieved from the Protein Data Bank (PDB ID 6JB1)^40^ and resolved residues 26–114 were used for subsequent modelling. This particular crystal structure of Kir6.2 was chosen for this study over other available structures of the channel because it has a good atomic resolution, the two residues focused of the study are resolved, and it was obtained in the presence of the SUR subunits. R50P mutant channels were generated using the Mutator plugin of VMD. N- and C-termini were acetylated and methylated, respectively. The VMD solvate plugin was used to solvate internal cavities of the protein. The structures were aligned perpendicular to the bilayer and inserted into a membrane, a neutral membrane containing 1-palmitoyl-2-oleoyl-sn-glycero-3-phosphocholine (POPC), with x and y dimensions of 96 Å. The VMD solvate plugin^60^ was then used to create a rectangular water box around the membrane-protein system. The overlapping water and lipid molecules around the ion channel structure were removed with the cut-off distance (1.2 Å). Potassium and chloride ions were added using Autoionize Plugin of VMD to neutralise the systems and obtain a concentration of 150 mM. The final system size was approximately 183,000 atoms.

MD simulations were performed with the gromacs v2020.1 software (https://doi.org/10.5281/zenodo.3562512). Four replicates of 500 ns were run for the wild type and R50P mutant amounting to 4 µs in total. CHARMM36^61^ parameters were used for the protein, ions, ATP and lipids, and the TIP3P model was used for water. The particle mesh Ewald method was used for the treatment of periodic electrostatic interactions^62^, with an upper threshold of 1 Å for grid spacing. Electrostatic and van der Waals forces were calculated every time step. A cut-off distance of 12 10 Å was used for van der Waals forces. A switching distance of 10 Å was chosen to smoothly truncate the non-bonded interactions. Only atoms in a Verlet pair list with a cut-off distance of 13.5 Å reassigned every 20 steps were considered. The SETTLE LINCS algorithm^63^ was used to constrain all bonds involving hydrogen atoms, to allow the use of a 2 fs time step throughout the simulation. MD simulations were performed in the NPT ensemble. The Parrinello–Rahman barostat^64^ was employed to control the pressure with a 50 fs period, and the desired value of 1 atmosphere. The system was coupled to a Nose–Hoover thermostat^65,66^ to sustain a temperature of 310.15 K throughout.

The same equilibration protocol was applied to all the systems. The systems were subjected to 10,000 steps of minimization, with harmonic constraints on protein backbone atoms and protein sidechains (20 kcal/mol/Å^2^), the ATP (force constant 40 kcal/mol/Å^2^), and lipid headgroups (10 kcal/mol/Å^2^). Harmonic restraints were gradually reduced to a force constant of 2 kcal/mol/Å^2^ and were removed in consecutive steps from the lipid headgroups, protein sidechains and protein backbone over the course of a 10 ns trajectory. Production runs followed and analysis of the simulations was performed with python v3.7.3 scripts using the MDTraj v1.9.4 python package.

### Electrophysiology

Electrophysiological analyses were performed according to the previous study^67^. Following incubation for 48 h after medium change to differentiation medium, 10 μL of AAV vectors carrying Kir6.2/WT or Kir6.2/R50P were added to the cultured dishes. Subsequently, after infection for 72 h, the medium was replaced with fresh medium, and incubated for an additional 5–6 days. All electrophysiological measurements were performed at room temperature (22–25 °C) using an EPC-800 patch clamp amplifier (HEKA Electronics, Lambrecht/Pfalz, Germany) and pCLAMP 10 software (Molecular Devices, CA, USA). The membrane potentials were recorded in the current clamp mode in standard whole-cell technique^68^ and the effects of 10 mM ACh and 0.1 mM glibenclamide were evaluated. The pipette solution contained (in mM): 107 KCl, 2 MgCl_2_, 1 CaCl_2_, 10 EGTA, and 10 HEPES at pH 7.2 with KOH. The extracellular solution contained (in mM): 138 NaCl, 5.6 KCl, 1 MgCl_2_, 10 HEPES, and 2.6 CaCl_2_ at pH 7.4 with NaOH. Standard patch clamp techniques with inside-out membrane patch was used to record K_ATP_ channel currents for MgATP sensitivity measurements. The pipette solution contained (in mM): 140 KCl, 2.0 CaCl_2_, 5.0 HEPES at pH 7.4 with KOH. The composition of bathing solution was (in mM): 110 KCl, 11 HEPES, 2 MgCl_2_, 11 EGTA, 1 CaCl_2_ at pH 7.2 with KOH. For the K_ATP_ channel recording, patch membrane potential was held at − 60 mV and various concentration of MgATP was applied. Relative channel activity was plotted as a function of MgATP concentrations, and each points were fitted in following equation.

$${\text{G}}/{\text{Gc}} = {1}/\left\{ {{1} + \left( {\left[ {{\text{MgATP}}} \right]/{\text{IC}}_{{{5}0}} } \right)^{{\text{h}}} } \right\}$$

where [MgATP] is the MgATP concentration, IC_50_ is the [MgATP] at which inhibition is half-maximal and *h* is the slope factor (Hill coefficient). Data were analyzed using Origin Graphing and Data Analysis Software (Version 9, OriginLab Corporation, MA; https://www.originlab.com).

### Criteria for [Ca^2+^]_i_ responses

[Ca^2+^]_i_ was measured as previously reported^69^. Mouse skeletal myotubes were incubated with 2 μmol/L Fura-2/AM (Dojin Chemical, Kumamoto, Japan) for 30 min at room temperature, mounted in a chamber and superfused with KRB at 1 mL/min at 37 °C. Fluorescence images due to excitation at 340 and 380 nm were captured and the ratio (F340/F380) images were produced by an Argus-50 system (Hamamatsu Photonics, Hamamatsu, Japan). Amplitudes of [Ca^2+^]_i_ responses to ACh were calculated by subtracting the prestimulatory basal [Ca^2+^]_i_ ratio from the peak [Ca^2+^]_i_ ratio. A response was considered when increases in [Ca^2+^]_i_ took place within 5 min after the addition of agents. For calculation of 10 mM of ACh, the basal [Ca^2+^]_i_ ratio from peak [Ca^2+^]_i_ ratio for 5 min within 10 min from onset of measurement was analyzed.

### Histological assessment

Following incubation for 48 h after medium change to differentiation medium, AAV vectors carrying Kir6.2/WT or Kir6.2/R50P were added to the cultured dishes. After infection for 72 h, the medium was replaced with fresh medium, and incubated for an additional 24 h. After removing the incubation media, the C2C12 cells were washed in cold PBS fixed in 100% methanol. The DAPI-containing media were mounted on a coating dish for confocal laser-scanning microscope (Fluoview FV10i; Olympus, Tokyo, Japan) and used to allow clear visualization of nuclei and myotube structures for quantitative measurements; the dishes were then covered with a cover glass. The myogenic index was used as a morphological parameter of muscle differentiation^70^. The viral infected myoblasts and myotobes were also confirmed by RFP fluorescence automatically cleaved from Kir6.2/WT or Kir6.2/R50P inside the cytosol. The number of nuclei in each myotube containing ≥ 3 nuclei and the total number of nuclei were counted in 30 randomly selected fields per well. The myogenic index (in %) was then calculated as: ([number of nuclei in myotubes]/[total number of nuclei in 30 randomly selected fields]) using ImageJ software version 1.51 (National Institutes of Health, Bethesda, MD, USA; https://imagej.nih.gov/ij/download.html). Myotube widths were measured by using a confocal laser-scanning microscope or the ImageJ software. In brief, cells were evaluated in 30 randomly selected fields per well. The width of each myotube containing ≥ 3 nuclei was measured on the cell, and the average width per myotube was calculated. A total of 60–90 myotubes was evaluated for each treatment group.

### Glucose uptake

For quantitative analyses, the glucose uptake was evaluated by fluorescent glucose analog, 2-[*N*-(7-nitrobenz-2-oxa-1,3-diazol-4-yl)amino]-2-deoxy-d-glucose (2-NBDG) uptake. After confirming the differentiation, the culture medium was changed to modified Krebs Ringer Buffer without glucose (20 mM HEPES pH 7.4, 135 mM NaCl, 5 mM KCl, 1 mM MgSO_4_, 0.4 mM K_2_HPO_4_, 1 mM CaCl_2_) and incubated for two hours. A final concentration of 50 μM 2-NBDG was added to the media, and incubated for two hours. After washing three times with PBS buffer, the dishes were covered with cover glasses. We confirmed that the results of 2-NBDG uptake showed a good correlation with 2-[18F]-2-deoxy-d-glucose (2-FDG) uptake, a well-established method for measurement of glucose uptake. A total of 60–90 myotubes (30 fields for each independent experiment) were evaluated for each treatment group.

### qRT-PCR and immunohistochemical analyses

Polymerase chain reaction amplification was performed by using a StepOne Real-time PCR system (Life Technologies, CA, USA) under the following conditions: one cycle of 95 °C for 30 s; 45 cycles of 95 °C for 5 s and 56 °C for 10 s and 72 °C for 15 s. The amplified fragments were applied to 2% (w/v) agarose gel, and visualized by staining with SYBR Safe DNA staining reagent (Life Technologies, CA, USA). In immunostaining experiments, the glass bottom dishes, which were cultured transfected and non-transfected myotubes, were washed in phosphate buffered saline (PBS) and incubated in blocking solution containing 2% bovine serum albumin (BSA) and 5% normal goat serum for 1 h. Then, the dishes were incubated with rabbit polyclonal Kir6.2 antibody (1:1000, ab81121, abcam, Cambridge, UK) for 48 h at 4 °C. Dishes were washed in PBS and incubated with Alexa 488-labelled anti-rabbit IgG (Invitrogen, CA, USA) for 30 min and mounted with a DAPI-containing mount medium and covered with a cover glass.

### Statistical analysis

As previously reported^71^, the data are presented as the means ± standard error of the mean (SEM) unless otherwise indicated. One-way analysis of variance (ANOVA) was used, and the treatment groups were compared with Tukey’s post hoc test for honest significant difference. All statistical analyses were performed using GraphPad Prism 8.2.1 (GraphPad Software, Inc., San Diego, CA, USA; https://www.graphpad.com/scientific-software/prism/) for MacOS, version 10.14.6. A *p* value of < 0.05 was considered statistically significant.

## Supplementary Information

Supplementary Information

## Abbreviations

K_ATP_: ATP-sensitive potassium channel
DEND: Developmental delay, epilepsy, and neonatal diabetes
ACh: Acetylcholine
2-NBDG: 2-[*N*-(7-Nitrobenz-2-oxa-1,3-diazol-4-yl)amino]-2-deoxy-d-glucose
