# Supplementary material for: Structure based analysis of KATP channel with a DEND syndrome mutation in murine skeletal muscle
Source: Sci Rep. 2021 Mar 23;11:6668. doi: 10.1038/s41598-021-86121-5 (PMC7988048; doi:10.1038/s41598-021-86121-5)
Supplement: Supplementary file 1 — Supplementary Information [file 41598_2021_86121_MOESM1_ESM.docx]

**Supplementary Material**

**Structure based analysis of K_ATP_ channel with a DEND syndrome mutation in murine skeletal muscle**

Shoichiro Horita^a^, Tomoyuki Ono^a^, Saul Gonzalez-Resines^b^, Yuko Ono^a^, Megumi Yamachi^a^, Songji Zhao^c^, Carmen Domene^b,d^, Yuko Maejima^a^, Kenju Shimomura^a^

*^a^Department of Bioregulation and Pharmacological Medicine, Fukushima Medical University School of Medicine, Fukushima 960-1295, Japan*

*^b^Department of Chemistry, University of Bath, Claverton Down, Bath, BA2 7AY, United Kingdom*

*^c^Advanced Clinical Research Center, Fukushima Global Medical Science Center, Fukushima Medical University, Japan*

*^d^Chemistry Research Laboratory, Mansfield Road, University of Oxford, Oxford OX1 3TA, United Kingdom*

**Supplementary Table 1.**

Primers used for the quantitative reverse transcription PCR reaction. eKir6.2 and vKir6.2 represent the primers targeting the endogenous Kir6.2 and the virally expressed Kir6.2, respectively.

|  | sense | antisense |
| --- | --- | --- |
| GAPDH | GAGAATTCATGGTGAAGGTCGGTGTGAA | GCCTCGAGTTACTCCTTGGAGGCCATGT |
| eKir6.2 | CAGAATATCGTCGGGCTGAT | CTCTTTCGGAGGTCCCCTAC |
| vKir6.2 | GTGATACTGGAAGGCGTGGT | CGAGAACTAGCCAGGGTCAG |
| SUR1 | GGAAGGACTCACCACCATC | GAGACCATCAAGGCGTAGG |
| SUR2A | GATGCCACTGTCACCGAAG | TCATCACAATAACCAGGTCTGC |
| MyoD | CGCGCTCCAACTGCTCTGATGG | CTCGACACAGCCGCACTCTTCC |
| Baf60c | AGGCTTACATGGACCTCCTAG | CATCAGAGTCTTCCGCATCAG |
| Deptor | GACGGCGATAAAACTCATGCA | CCTTGTGCTCATCACACACGT |
| AKT2 | GTGATGCGAAGGAGGTCAT | TGCTTGTGTCCTGTGGTG |

**Supplementary Fig. 1.** Quantitative reverse transcription PCR measurements of the expression of viral Kir6.2 (vKir6.2) (a), endogenous Kir6.2 (eKir6.2) (b), SUR1 (c), SUR2A (d), MyoD (e), Baf60c (f), Deptor (g), AKT2 (h) (N=4).

**
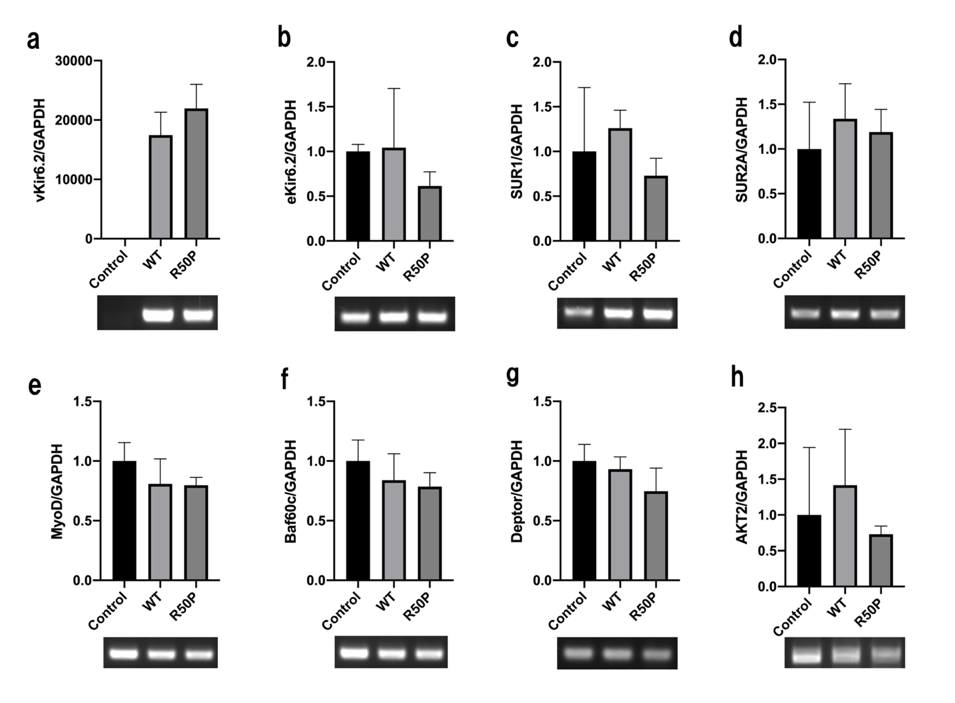
**

**Supplementary Fig. 2.** Fluorescence microscopy images of the control (non transfected), WT-, and R50P-transfected myotubes. Green fluorescence represent immunostaining with Kir6.2 antibodies for both endogenous and virally expressed Kir6.2. Viral infection was also confirmed by red florescence.


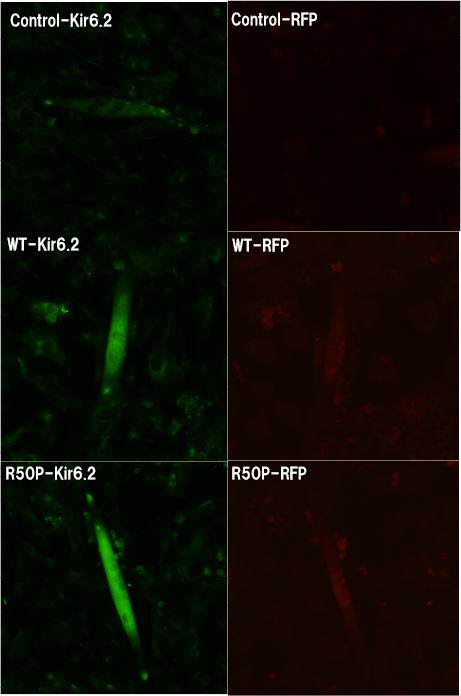
 **Supplementary Fig. 3.** Quantitative reverse transcription PCR measurements of the expression of endogenous Kir6.2 and SUR2A, before and after differentiation (N=4-5) (*P<0.05). The used primers are shown in Supplementary Table S1.


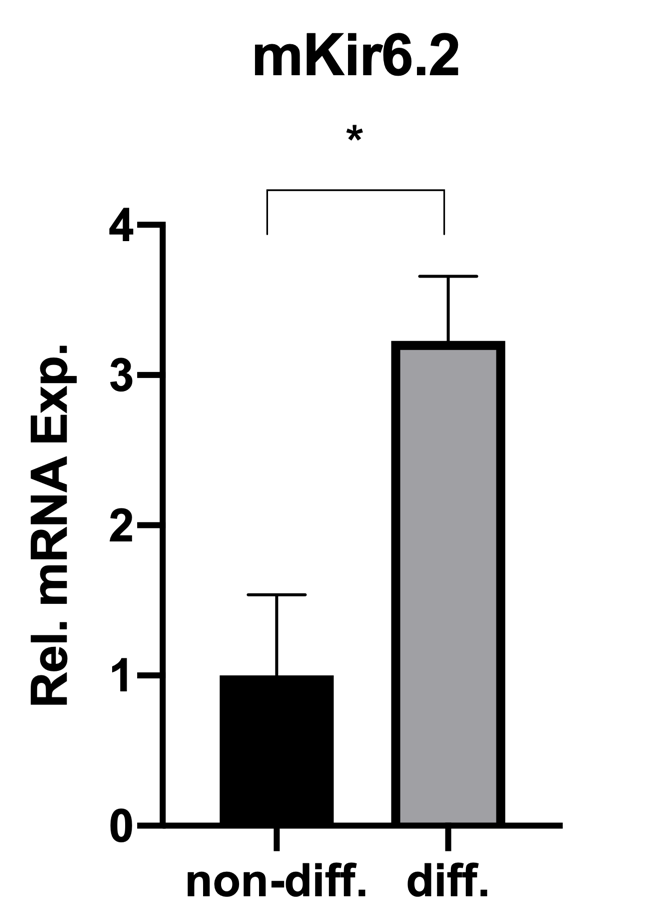

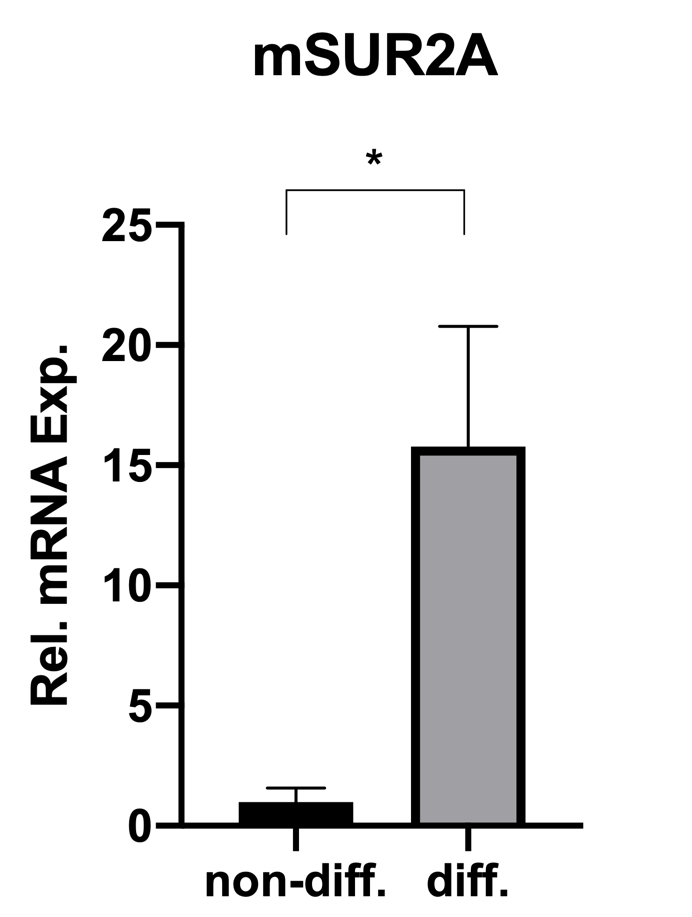


**Supplementary Fig. 4.** Mean membrane potential (± SEM) of C2C12 myoblast without infection (n = 9), with infection of the Kir6.2/WT AAV vector (n = 7), the Kir6.2/R50P AAV vector (n = 7). Multiple groups were compared using an ordinary One-Way ANOVA test, followed by Tukey’s test. (*P < 0.05).

**
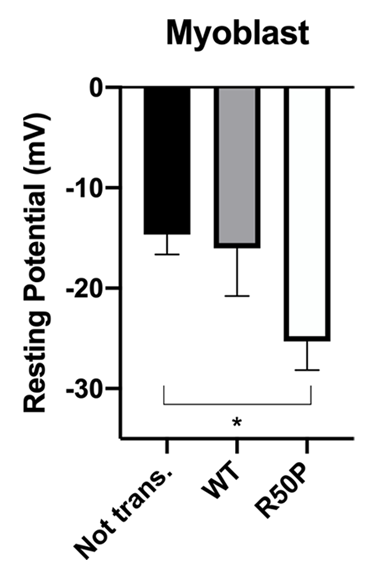
**

**Supplementary Fig. 5.** Evolution of the Cα root-mean-square deviation (RMSD) of the channel structure computed for the trajectories taking as a reference the initial cryoEM structure. (a) Analysis of the Cα RMSD values for the entire tetramer, (b) M1helix (residues 67-97) and (c) M2 helix (residues 142-172). The overall RMSD values are the same for all the simulations suggesting overall conformational stability of the protein at these timescales with respect to the initial cryoEM structure.

| **(a)** | **(b)** | **(c)** |
| --- | --- | --- |
| 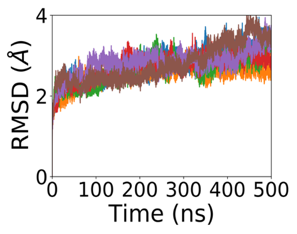 | 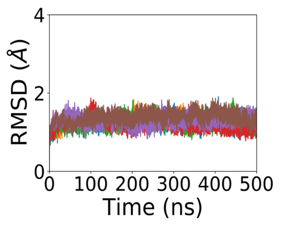 | 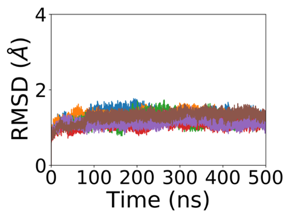 |
|  | | |

**Supplementary Fig. 6.** Representative interactions between Kir6.2 and ATP in the two systems considered in this study: wild type and the R50P mutant. (A) Notation used for the names of the ATP and amino acid atoms involved in the interactions considered. (B) Representative interactions are displayed for the four monomers and the corresponding ATP molecule. ** indicates that the interactions are absent or become weaker as the simulation progresses. In brackets is the number of monomers where the interactions are found. “X” represents “not recorded”.

| **(A)** | | | |
| --- | --- | --- | --- |
| **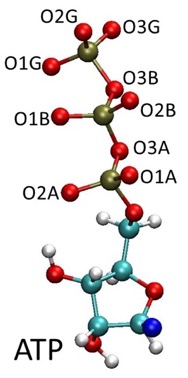** | **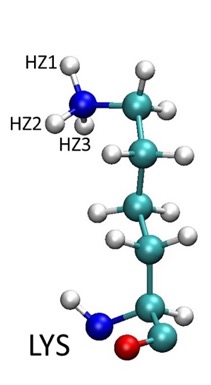** | **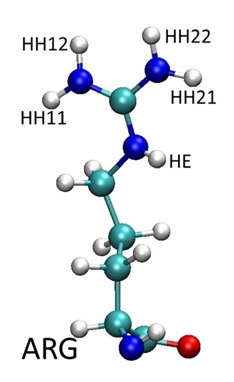** | **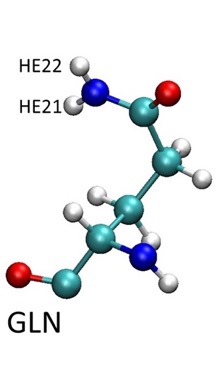** |

| **(B)** | | |
| --- | --- | --- |
| **Wild type: Lys185(4)/Arg54(2)/Arg50(4)/Lys39(4)** | | |
| **Monomer 1** | | |
| Lys185 HZ1 – ATP O1A | Lys185 HZ2 – ATP O1A | Lys185 HZ3 – ATP O1A |
| 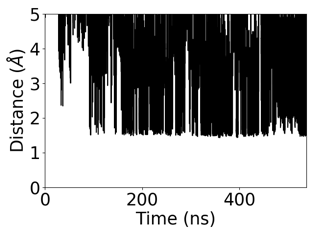 | 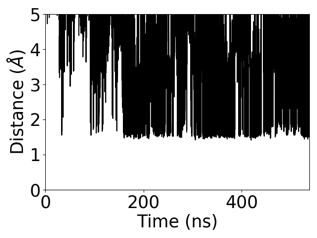 | 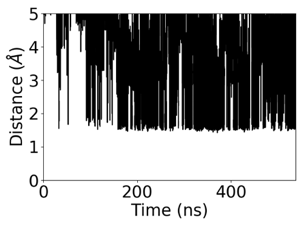 |
| **Lys185 HZ1 – ATP O1B | **Lys185 HZ2 – ATP O1B | **Lys185 HZ3 – ATP O1B |
| 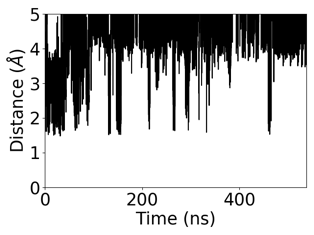 | 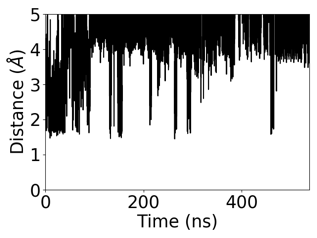 | 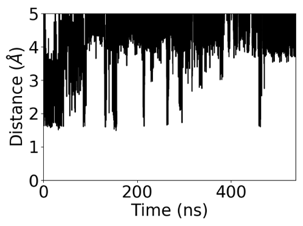 |
| **Lys185 HZ1 – ATP O1G | **Lys185 HZ2 – ATP O1G | **Lys185 HZ3 – ATP O1G |
| 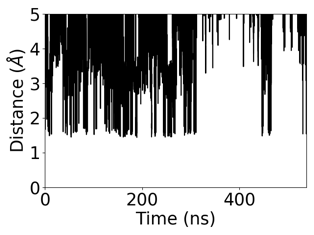 | 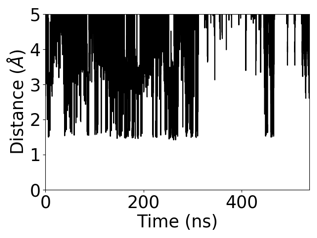 | 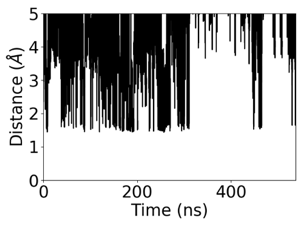 |
| **Lys185 HZ1 – ATP O2A | **Lys185 HZ2 – ATP O2A | **Lys185 HZ3 – ATP O2A |
| 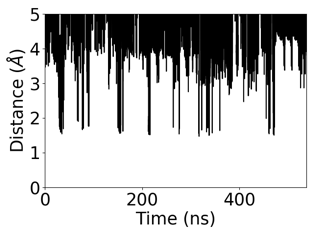 | 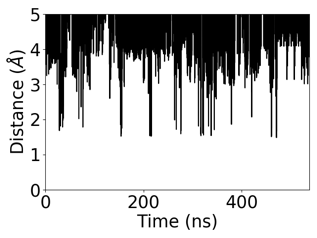 | 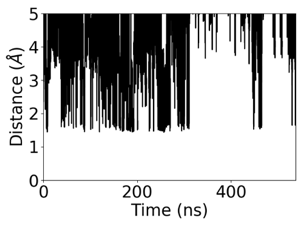 |
| Lys185 HZ1 – ATP O2B | Lys185 HZ2 – ATP O2B | Lys185 HZ3 – ATP O2B |
| 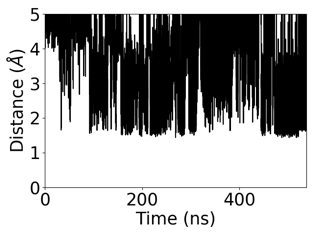 | 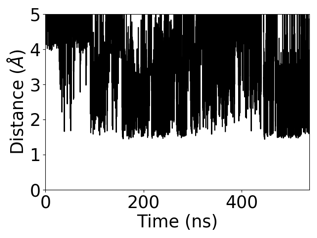 | 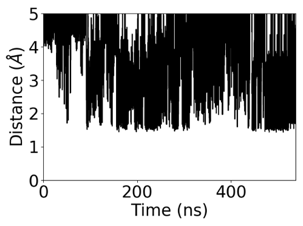 |
| **Lys185 HZ1 – ATP O2G | **Lys185 HZ2 – ATP O2G | **Lys185 HZ3 – ATP O2G |
| 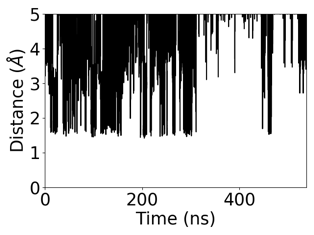 | 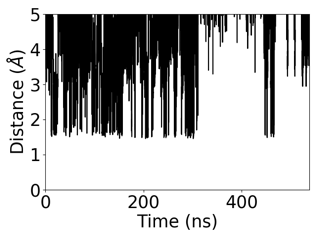 | 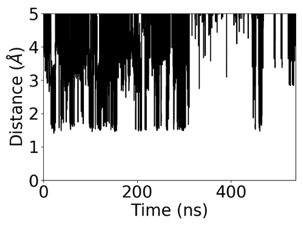 |
| **Lys185 HZ1 – ATP O3A | **Lys185 HZ2 – ATP O3A | **Lys185 HZ3 – ATP O3A |
| 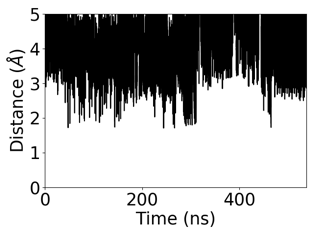 | 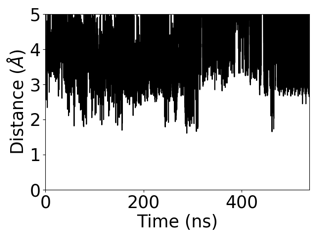 | 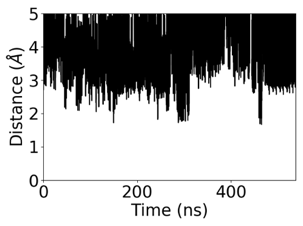 |
| **Lys185 HZ1 – ATP O3B | **Lys185 HZ2 – ATP O3B | **Lys185 HZ3 – ATP O3B |
| 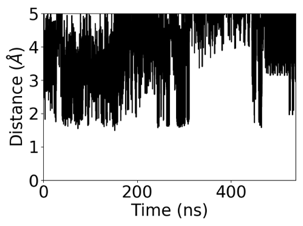 | 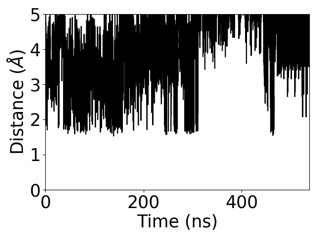 | 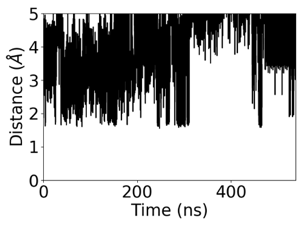 |
| **Lys185 HZ1 – ATP O3G | **Lys185 HZ2 – ATP O3G | **Lys185 HZ3 – ATP O3G |
| 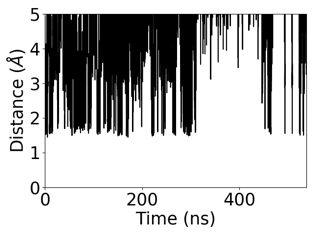 | 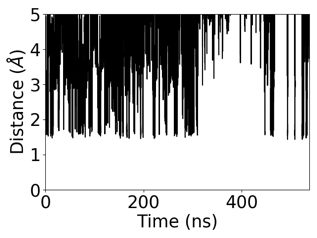 | 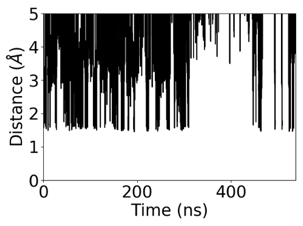 |
| Arg50 HH11 – ATP O1A | **Arg50 HH12 – ATP O1A |  |
| X | 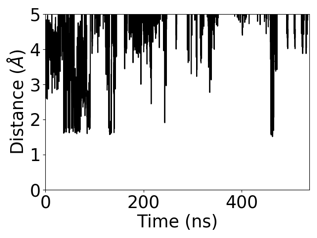 |  |
| Arg50 HH11 – ATP O1B | Arg50 HH12 – ATP O1B |  |
| 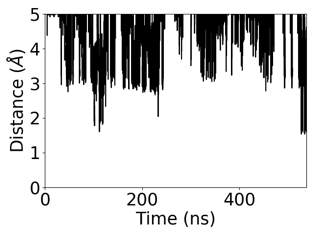 | 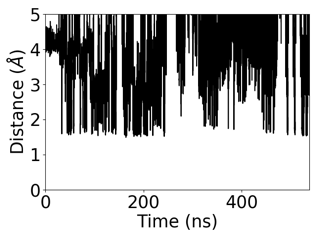 |  |
| Arg50 HH11 – ATP O1G | Arg50 HH12 – ATP O1G |  |
| X | 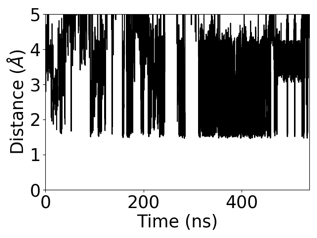 |  |
| Arg50 HH21 – ATP O1A | **Arg50 HH22 – ATP O1A |  |
| X | 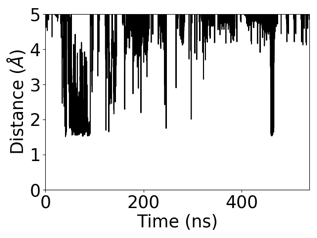 |  |
| Arg50 HH21 – ATP O1B | Arg50 HH22 – ATP O1B |  |
| X | 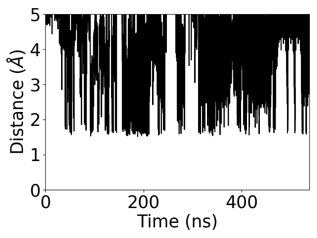 |  |
| Arg50 HH21 – ATP O1G | Arg50 HH22 – ATP O1G |  |
| X | 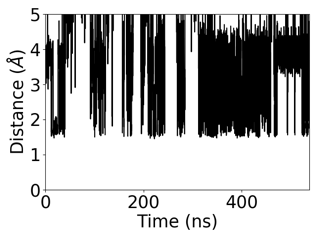 |  |
| Arg50 HH11 – ATP O2A | Arg50 HH12 – ATP O2A |  |
| X | 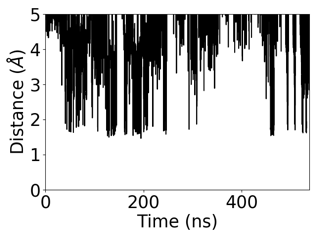 |  |
| Arg50 HH11 – ATP O2B | **Arg50 HH12 – ATP O2B |  |
| X | 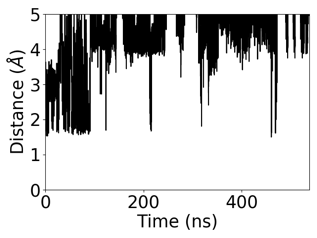 |  |
| Arg50 HH11 – ATP O2G | Arg50 HH12 – ATP O2G |  |
| X | 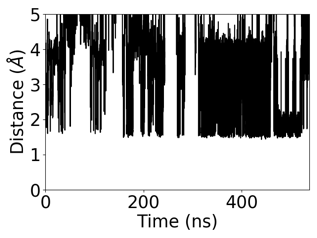 |  |
| Arg50 HH21 – ATP O2A | Arg50 HH22 – ATP O2A |  |
| X | 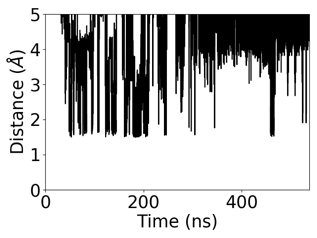 |  |
| Arg50 HH21 – ATP O2B | **Arg50 HH22 – ATP O2B |  |
| X | 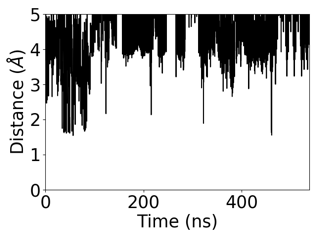 |  |
| Arg50 HH21 – ATP O2G | Arg50 HH22 – ATP O2G |  |
| X | 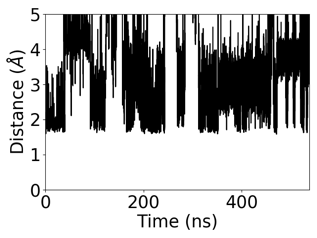 |  |
| Arg50 HH11 – ATP O3A | Arg50 HH12 – ATP O3A |  |
| X | 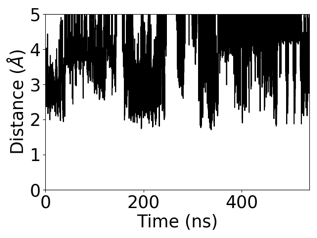 |  |
| Arg50 HH11 – ATP O3B | Arg50 HH12 – ATP O3B |  |
| X | 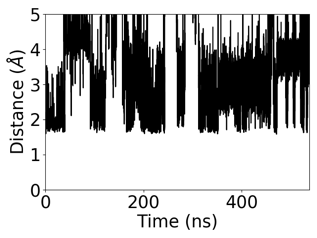 |  |
| Arg50 HH11 – ATP O3G | Arg50 HH12 – ATP O3G |  |
| X | 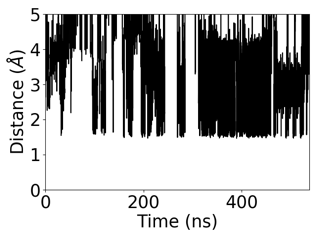 |  |
| Arg50 HH21 – ATP O3A | Arg50 HH22 – ATP O3A |  |
| X | 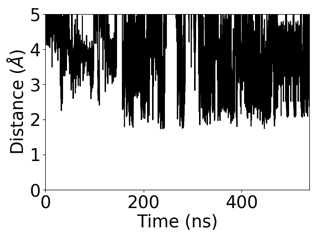 |  |
| Arg50 HH21 – ATP O3B | Arg50 HH22 – ATP O3B |  |
| X | 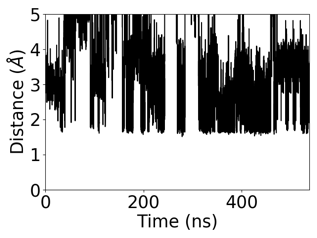 |  |
| Arg50 HH21 – ATP O3G | Arg50 HH22 – ATP O3G |  |
| X | 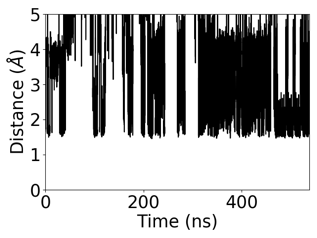 |  |
| Lys39 HZ1 - ATP O1A | Lys39 HZ2 - ATP O1A | Lys39 HZ3 - ATP O1A |
| X | X | X |
| Lys39 HZ1 - ATP O1B | Lys39 HZ2 - ATP O1B | Lys39 HZ3 - ATP O1B |
| X | X | X |
| **Lys39 HZ1 - ATP O1G | **Lys39 HZ2 - ATP O1G | **Lys39 HZ3 - ATP O1G |
| 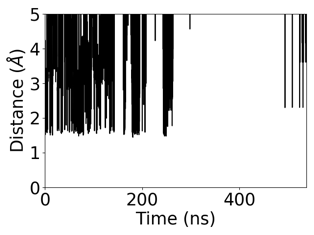 | 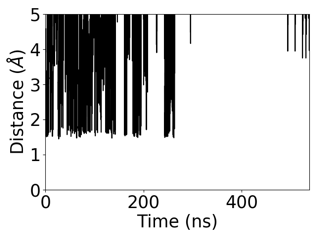 | 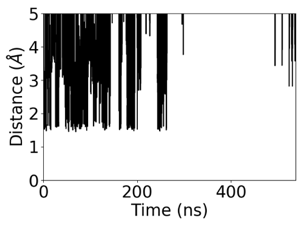 |
| Lys39 HZ1 - ATP O2A | Lys39 HZ2 - ATP O2A | Lys39 HZ3 - ATP O2A |
| X | X | X |
| Lys39 HZ1 - ATP O2B | Lys39 HZ2 - ATP O2B | Lys39 HZ3 - ATP O2B |
| X | X | X |
| **Lys39 HZ1 - ATP O2G | **Lys39 HZ2 - ATP O2G | **Lys39 HZ3 - ATP O2G |
| 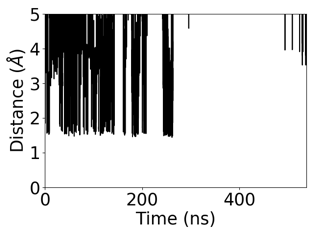 | 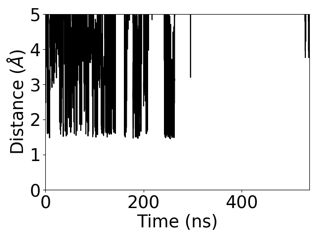 | 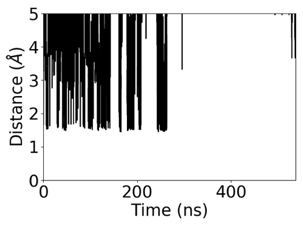 |
| Lys39 HZ1 - ATP O3A | Lys39 HZ2 - ATP O3A | Lys39 HZ3 - ATP O3A |
| X | X | X |
| Lys39 HZ1 - ATP O3B | Lys39 HZ2 - ATP O3B | Lys39 HZ3 - ATP O3B |
| X | X | X |
| **Lys39 HZ1 - ATP O3G | **Lys39 HZ2 - ATP O3G | **Lys39 HZ3 - ATP O3G |
| 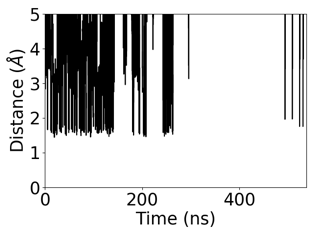 | 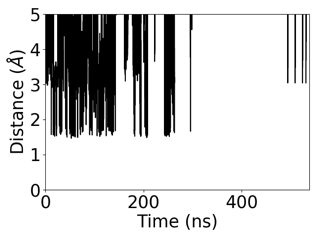 | 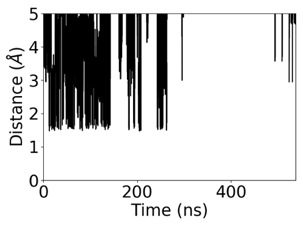 |
| **Monomer 2** | | |
| Lys185 HZ1 – ATP O1A | Lys185 HZ2 – ATP O1A | Lys185 HZ3 – ATP O1A |
| 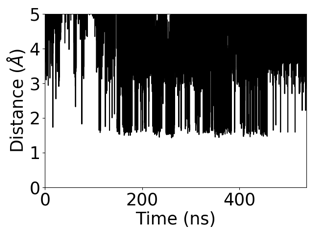 | 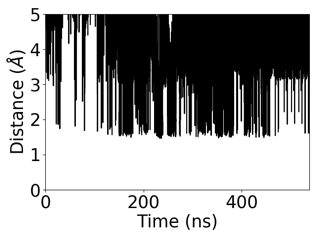 | 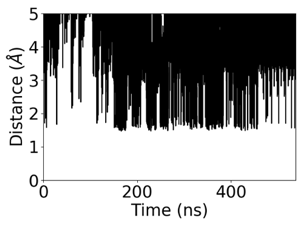 |
| **Lys185 HZ1 – ATP O1B | **Lys185 HZ2 – ATP O1B | **Lys185 HZ3 – ATP O1B |
| 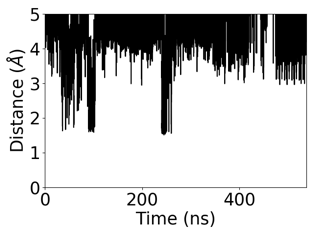 | 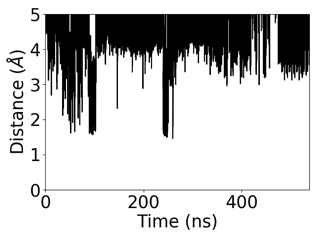 | 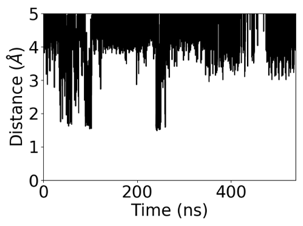 |
| Lys185 HZ1 – ATP O1G | Lys185 HZ2 – ATP O1G | Lys185 HZ3 – ATP O1G |
| 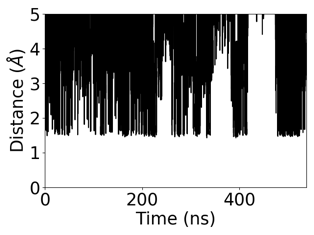 | 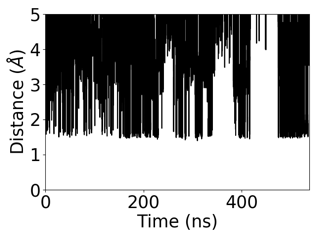 | 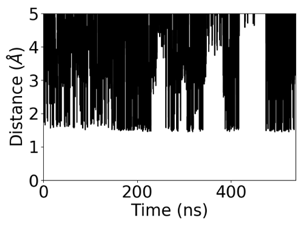 |
| Lys185 HZ1 – ATP O2A | Lys185 HZ2 – ATP O2A | Lys185 HZ3 – ATP O2A |
| X | X | X |
| Lys185 HZ1 – ATP O2B | Lys185 HZ2 – ATP O2B | Lys185 HZ3 – ATP O2B |
| 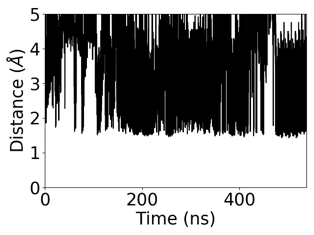 | 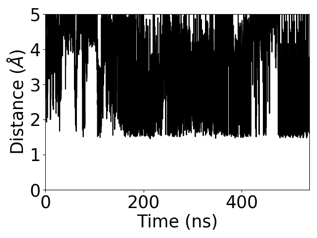 | 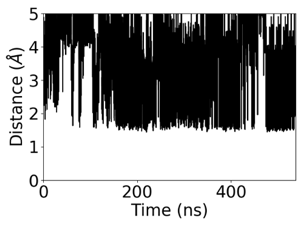 |
| Lys185 HZ1 – ATP O2G | Lys185 HZ2 – ATP O2G | Lys185 HZ3 – ATP O2G |
| 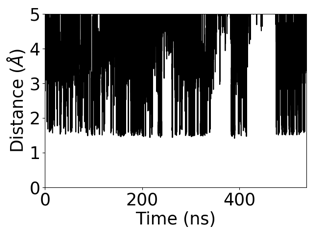 | 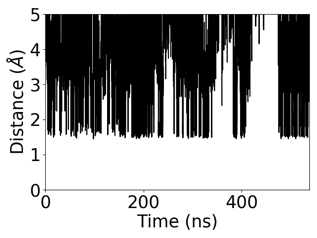 | 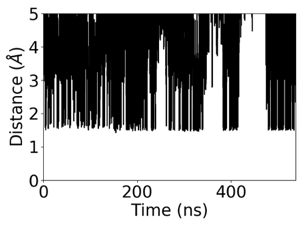 |
| **Lys185 HZ1 – ATP O3A | **Lys185 HZ2 – ATP O3A | **Lys185 HZ3 – ATP O3A |
| 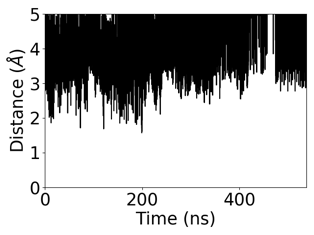 | 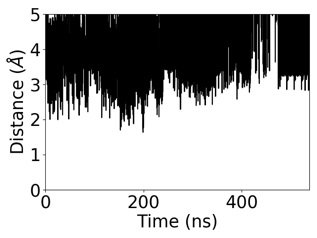 | 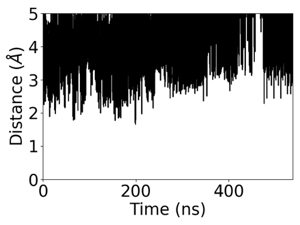 |
| Lys185 HZ1 – ATP O3B | Lys185 HZ2 – ATP O3B | Lys185 HZ3 – ATP O3B |
| 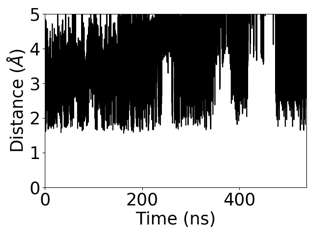 | 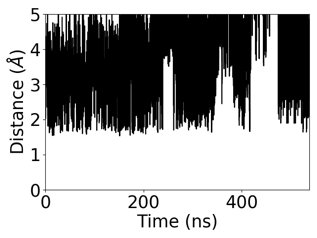 | 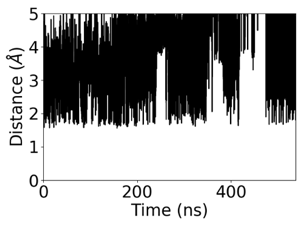 |
| Lys185 HZ1 – ATP O3G | Lys185 HZ2 – ATP O3G | Lys185 HZ3 – ATP O3G |
| 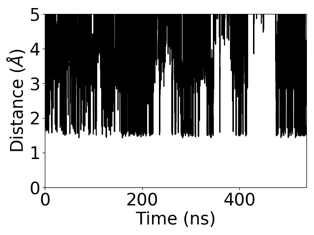 | 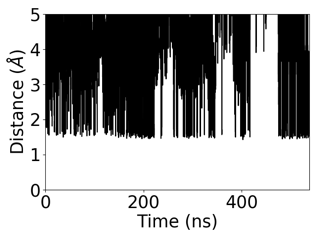 | 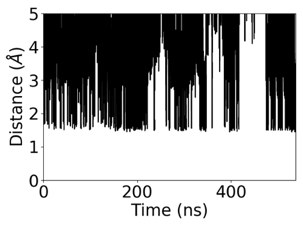 |
| Arg50 HH11 – ATP O1A | Arg50 HH12 – ATP O1A |  |
| X | X |  |
| Arg50 HH11 – ATP O1B | Arg50 HH12 – ATP O1B |  |
| X | 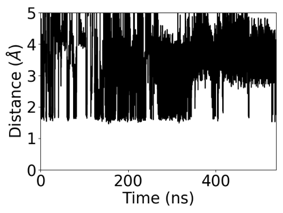 |  |
| Arg50 HH11 – ATP O1G | Arg50 HH12 – ATP O1G |  |
| X | 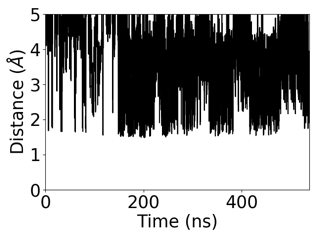 |  |
| Arg50 HH21 – ATP O1A | Arg50 HH22 – ATP O1A |  |
| X | 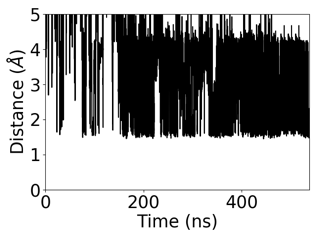 |  |
| Arg50 HH21 – ATP O1B | Arg50 HH22 – ATP O1B |  |
| X | 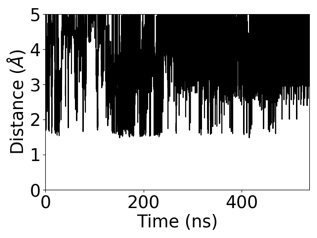 |  |
| Arg50 HH21 – ATP O1G | Arg50 HH22 – ATP O1G |  |
| X | X |  |
| Arg50 HH11 – ATP O2A | Arg50 HH12 – ATP O2A |  |
| 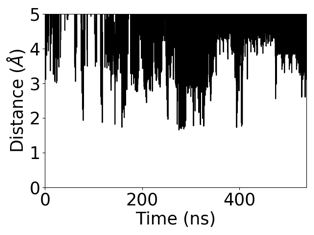 | 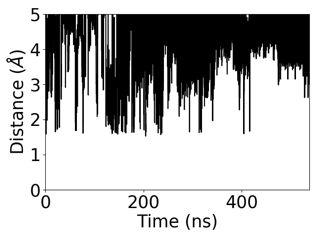 |  |
| Arg50 HH11 – ATP O2B | Arg50 HH12 – ATP O2B |  |
| X | X |  |
| Arg50 HH11 – ATP O2G | Arg50 HH12 – ATP O2G |  |
| X | 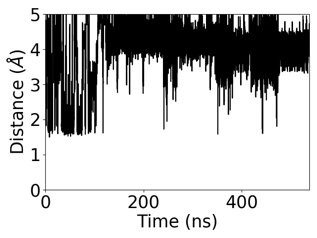 |  |
| Arg50 HH21 – ATP O2A | Arg50 HH22 – ATP O2A |  |
| X | 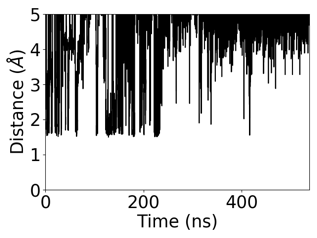 |  |
| Arg50 HH21 – ATP O2B | Arg50 HH22 – ATP O2B |  |
| X | 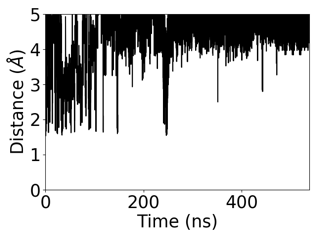 |  |
| Arg50 HH21 – ATP O2G | Arg50 HH22 – ATP O2G |  |
| X |  |  |
| Arg50 HH11 – ATP O3A | Arg50 HH12 – ATP O3A |  |
|  |  |  |
| Arg50 HH11 – ATP O3B | Arg50 HH12 – ATP O3B |  |
| X |  |  |
| Arg50 HH11 – ATP O3G | Arg50 HH12 – ATP O3G |  |
| X |  |  |
| Arg50 HH21 – ATP O3A | Arg50 HH22 – ATP O3A |  |
| X |  |  |
| Arg50 HH21 – ATP O3B | Arg50 HH22 – ATP O3B |  |
| X |  |  |
| Arg50 HH21 – ATP O3G | Arg50 HH22 – ATP O3G |  |
| X |  |  |
| Lys39 HZ1 - ATP O1A | Lys39 HZ2 - ATP O1A | Lys39 HZ3 - ATP O1A |
| X | X | X |
| Lys39 HZ1 - ATP O1B | Lys39 HZ2 - ATP O1B | Lys39 HZ3 - ATP O1B |
| X | X | X |
| Lys39 HZ1 - ATP O1G | Lys39 HZ2 - ATP O1G | Lys39 HZ3 - ATP O1G |
|  |  |  |
| Lys39 HZ1 - ATP O2A | Lys39 HZ2 - ATP O2A | Lys39 HZ3 - ATP O2A |
| X | X | X |
| **Lys39 HZ1 - ATP O2B | **Lys39 HZ2 - ATP O2B | **Lys39 HZ3 - ATP O2B |
|  |  |  |
| Lys39 HZ1 - ATP O2G | Lys39 HZ2 - ATP O2G | Lys39 HZ3 - ATP O2G |
|  |  |  |
| Lys39 HZ1 - ATP O3A | Lys39 HZ2 - ATP O3A | Lys39 HZ3 - ATP O3A |
| X | X | X |
| Lys39 HZ1 - ATP O3B | Lys39 HZ2 - ATP O3B | Lys39 HZ3 - ATP O3B |
| X | X | X |
| Lys39 HZ1 - ATP O3G | Lys39 HZ2 - ATP O3G | Lys39 HZ3 - ATP O3G |
|  |  |  |
| **Monomer 3** | | |
| Lys185 HZ1 – ATP O1A | Lys185 HZ2 – ATP O1A | Lys185 HZ3 – ATP O1A |
|  |  |  |
| **Lys185 HZ1 – ATP O1B | **Lys185 HZ2 – ATP O1B | **Lys185 HZ3 – ATP O1B |
|  |  |  |
| Lys185 HZ1 – ATP O1G | Lys185 HZ2 – ATP O1G | Lys185 HZ3 – ATP O1G |
|  |  |  |
| Lys185 HZ1 – ATP O2A | Lys185 HZ2 – ATP O2A | Lys185 HZ3 – ATP O2A |
|  |  |  |
| Lys185 HZ1 – ATP O2B | Lys185 HZ2 – ATP O2B | Lys185 HZ3 – ATP O2B |
|  |  |  |
| Lys185 HZ1 – ATP O2G | Lys185 HZ2 – ATP O2G | Lys185 HZ3 – ATP O2G |
|  |  |  |
| Lys185 HZ1 – ATP O3A | Lys185 HZ2 – ATP O3A | Lys185 HZ3 – ATP O3A |
| X |  |  |
| Lys185 HZ1 – ATP O3B | Lys185 HZ2 – ATP O3B | Lys185 HZ3 – ATP O3B |
|  |  |  |
| Lys185 HZ1 – ATP O3G | Lys185 HZ2 – ATP O3G | Lys185 HZ3 – ATP O3G |
|  |  |  |
| Arg54 HH11 – ATP O1A | Arg54 HH12 – ATP O1A |  |
| X | X |  |
| Arg54 HH11 – ATP O1B | Arg54 HH12 – ATP O1B |  |
|  |  |  |
| Arg54 HH11 – ATP O1G | Arg54 HH12 – ATP O1G |  |
|  | X |  |
| Arg54 HH21 – ATP O1A | Arg54 HH22 – ATP O1A |  |
| X | X |  |
| Arg54 HH21 – ATP O1B | Arg54 HH22 – ATP O1B |  |
| X | X |  |
| Arg54 HH21 – ATP O1G | Arg54 HH22 – ATP O1G |  |
| X |  |  |
| Arg54 HH11 – ATP O2A | Arg54 HH12 – ATP O2A |  |
| X | X |  |
| Arg54 HH11 – ATP O2B | Arg54 HH12 – ATP O2B |  |
|  | X |  |
| Arg54 HH11 – ATP O2G | Arg54 HH12 – ATP O2G |  |
|  | X |  |
| Arg54 HH21 – ATP O2A | Arg54 HH22 – ATP O2A |  |
| X | X |  |
| Arg54 HH21 – ATP O2B | Arg54 HH22 – ATP O2B |  |
| X |  |  |
| Arg54 HH21 – ATP O2G | Arg54 HH22 – ATP O2G |  |
| X |  |  |
| Arg54 HH11 – ATP O3A | Arg54 HH12 – ATP O3A |  |
|  | X |  |
| Arg54 HH11 – ATP O3B | Arg54 HH12 – ATP O3B |  |
|  | X |  |
| Arg54 HH11 – ATP O3G | Arg54 HH12 – ATP O3G |  |
|  | X |  |
| Arg54 HH21 – ATP O3A | Arg54 HH22 – ATP O3A |  |
| X |  |  |
| Arg54 HH21 – ATP O3B | Arg54 HH22 – ATP O3B |  |
| X |  |  |
| Arg54 HH21 – ATP O3G | Arg54 HH22 – ATP O3G |  |
| X |  |  |
| Arg50 HH11 – ATP O1A | Arg50 HH12 – ATP O1A |  |
| X | X |  |
| Arg50 HH11 – ATP O1B | Arg50 HH12 – ATP O1B |  |
| X |  |  |
| Arg50 HH11 – ATP O1G | Arg50 HH12 – ATP O1G |  |
| X |  |  |
| Arg50 HH21 – ATP O1A | Arg50 HH22 – ATP O1A |  |
| X | X |  |
| Arg50 HH21 – ATP O1B | Arg50 HH22 – ATP O1B |  |
| X |  |  |
| Arg50 HH21 – ATP O1G | Arg50 HH22 – ATP O1G |  |
| X |  |  |
| Arg50 HH11 – ATP O2A | Arg50 HH12 – ATP O2A |  |
| X |  |  |
| Arg50 HH11 – ATP O2B | Arg50 HH12 – ATP O2B |  |
| X | X |  |
| Arg50 HH11 – ATP O2G | Arg50 HH12 – ATP O2G |  |
| X |  |  |
| Arg50 HH21 – ATP O2A | Arg50 HH22 – ATP O2A |  |
|  |  |  |
| Arg50 HH21 – ATP O2B | **Arg50 HH22 – ATP O2B |  |
| X |  |  |
| Arg50 HH21 – ATP O2G | Arg50 HH22 – ATP O2G |  |
| X |  |  |
| Arg50 HH11 – ATP O3A | Arg50 HH12 – ATP O3A |  |
| X | X |  |
| Arg50 HH11 – ATP O3B | Arg50 HH12 – ATP O3B |  |
| X |  |  |
| Arg50 HH11 – ATP O3G | Arg50 HH12 – ATP O3G |  |
| X |  |  |
| Arg50 HH21 – ATP O3A | Arg50 HH22 – ATP O3A |  |
| X |  |  |
| Arg54 HH21 – ATP O3B | Arg50 HH22 – ATP O3B |  |
| X |  |  |
| Arg54 HH21 – ATP O3G | Arg54 HH22 – ATP O3G |  |
| X |  |  |
| Lys39 HZ1 - ATP O1A | Lys39 HZ2 - ATP O1A | Lys39 HZ3 - ATP O1A |
| X | X | X |
| Lys39 HZ1 - ATP O1B | Lys39 HZ2 - ATP O1B | Lys39 HZ3 - ATP O1B |
| X | X | X |
| Lys39 HZ1 - ATP O1G | Lys39 HZ2 - ATP O1G | Lys39 HZ3 - ATP O1G |
|  |  |  |
| Lys39 HZ1 - ATP O2A | Lys39 HZ2 - ATP O2A | Lys39 HZ3 - ATP O2A |
| X | X | X |
| **Lys39 HZ1 - ATP O2B | **Lys39 HZ2 - ATP O2B | **Lys39 HZ3 - ATP O2B |
|  |  |  |
| Lys39 HZ1 - ATP O2G | Lys39 HZ2 - ATP O2G | Lys39 HZ3 - ATP O2G |
| X | X | X |
| Lys39 HZ1 - ATP O3A | Lys39 HZ2 - ATP O3A | Lys39 HZ3 - ATP O3A |
| X | X | X |
| Lys39 HZ1 - ATP O3B | Lys39 HZ2 - ATP O3B | Lys39 HZ3 - ATP O3B |
| X | X | X |
| Lys39 HZ1 - ATP O3G | Lys39 HZ2 - ATP O3G | Lys39 HZ3 - ATP O3G |
|  |  |  |
| **Monomer 4** | | |
| Lys185 HZ1 – ATP O1A | Lys185 HZ2 – ATP O1A | Lys185 HZ3 – ATP O1A |
|  |  |  |
| **Lys185 HZ1 – ATP O1B | **Lys185 HZ2 – ATP O1B | **Lys185 HZ3 – ATP O1B |
|  |  |  |
| Lys185 HZ1 – ATP O1G | Lys185 HZ2 – ATP O1G | Lys185 HZ3 – ATP O1G |
|  |  |  |
| Lys185 HZ1 – ATP O2A | Lys185 HZ2 – ATP O2A | Lys185 HZ3 – ATP O2A |
|  |  |  |
| Lys185 HZ1 – ATP O2B | Lys185 HZ2 – ATP O2B | Lys185 HZ3 – ATP O2B |
|  |  |  |
| Lys185 HZ1 – ATP O2G | Lys185 HZ2 – ATP O2G | Lys185 HZ3 – ATP O2G |
|  |  |  |
| Lys185 HZ1 – ATP O3A | Lys185 HZ2 – ATP O3A | Lys185 HZ3 – ATP O3A |
| X | X | X |
| Lys185 HZ1 – ATP O3B | Lys185 HZ2 – ATP O3B | Lys185 HZ3 – ATP O3B |
|  |  |  |
| Lys185 HZ1 – ATP O3G | Lys185 HZ2 – ATP O3G | Lys185 HZ3 – ATP O3G |
|  |  |  |
| Arg54 HH11 – ATP O1A | Arg54 HH12 – ATP O1A |  |
| X | X |  |
| Arg54 HH11 – ATP O1B | Arg54 HH12 – ATP O1B |  |
|  | X |  |
| Arg54 HH11 – ATP O1G | Arg54 HH12 – ATP O1G |  |
|  | X |  |
| Arg54 HH21 – ATP O1A | Arg54 HH22 – ATP O1A |  |
|  | X |  |
| Arg54 HH21 – ATP O1B | Arg54 HH22 – ATP O1B |  |
| X |  |  |
| Arg54 HH21 – ATP O1G | Arg54 HH22 – ATP O1G |  |
|  |  |  |
| Arg54 HH11 – ATP O2A | Arg54 HH12 – ATP O2A |  |
| X | X |  |
| Arg54 HH11 – ATP O2B | Arg54 HH12 – ATP O2B |  |
| X | X |  |
| Arg54 HH11 – ATP O2G | Arg54 HH12 – ATP O2G |  |
|  | X |  |
| Arg54 HH21 – ATP O2A | Arg54 HH22 – ATP O2A |  |
| X | X |  |
| Arg54 HH21 – ATP O2B | Arg54 HH22 – ATP O2B |  |
|  |  |  |
| Arg54 HH21 – ATP O2G | Arg54 HH22 – ATP O2G |  |
|  |  |  |
| Arg54 HH11 – ATP O3A | Arg54 HH12 – ATP O3A |  |
| X | X |  |
| Arg54 HH11 – ATP O3B | Arg54 HH12 – ATP O3B |  |
|  | X |  |
| Arg54 HH11 – ATP O3G | Arg54 HH12 – ATP O3G |  |
|  | X |  |
| Arg54 HH21 – ATP O3A | Arg54 HH22 – ATP O3A |  |
| X |  |  |
| Arg54 HH21 – ATP O3B | Arg54 HH22 – ATP O3B |  |
|  |  |  |
| Arg54 HH21 – ATP O3G | **Arg54 HH22 – ATP O3G |  |
|  |  |  |
| Arg50 HH11 – ATP O1A | Arg50 HH12 – ATP O1A |  |
| X | X |  |
| Arg50 HH11 – ATP O1B | **Arg50 HH12 – ATP O1B |  |
| X |  |  |
| Arg50 HH11 – ATP O1G | **Arg50 HH12 – ATP O1G |  |
| X |  |  |
| Arg50 HH21 – ATP O1A | Arg50 HH22 – ATP O1A |  |
| X | X |  |
| **Arg50 HH21 – ATP O1B | **Arg50 HH22 – ATP O1B |  |
|  |  |  |
| Arg50 HH21 – ATP O1G | Arg50 HH22 – ATP O1G |  |
| X |  |  |
| Arg50 HH11 – ATP O2A | Arg50 HH12 – ATP O2A |  |
| X |  |  |
| Arg50 HH11 – ATP O2B | Arg50 HH12 – ATP O2B |  |
| X | X |  |
| Arg50 HH11 – ATP O2G | Arg50 HH12 – ATP O2G |  |
| X |  |  |
| Arg50 HH21 – ATP O2A | Arg50 HH22 – ATP O2A |  |
| X | X |  |
| Arg50 HH21 – ATP O2B | Arg50 HH22 – ATP O2B |  |
| X | X |  |
| Arg50 HH21 – ATP O2G | Arg50 HH22 – ATP O2G |  |
| X |  |  |
| Arg50 HH11 – ATP O3A | Arg50 HH12 – ATP O3A |  |
| X |  |  |
| Arg50 HH11 – ATP O3B | Arg50 HH12 – ATP O3B |  |
| X |  |  |
| Arg50 HH11 – ATP O3G | Arg50 HH12 – ATP O3G |  |
| X |  |  |
| Arg50 HH21 – ATP O3A | **Arg50 HH22 – ATP O3A |  |
| X |  |  |
| Arg54 HH21 – ATP O3B | Arg50 HH22 – ATP O3B |  |
| X | X |  |
| Arg54 HH21 – ATP O3G | Arg54 HH22 – ATP O3G |  |
| X |  |  |
| **Lys39 HZ1 - ATP O1A | **Lys39 HZ2 - ATP O1A | **Lys39 HZ3 - ATP O1A |
|  |  |  |
| **Lys39 HZ1 - ATP O1B | **Lys39 HZ2 - ATP O1B | **Lys39 HZ3 - ATP O1B |
|  |  |  |
| **Lys39 HZ1 - ATP O1G | **Lys39 HZ2 - ATP O1G | **Lys39 HZ3 - ATP O1G |
|  |  |  |
| **Lys39 HZ1 - ATP O2A | **Lys39 HZ2 - ATP O2A | **Lys39 HZ3 - ATP O2A |
|  |  |  |
| **Lys39 HZ1 - ATP O2B | **Lys39 HZ2 - ATP O2B | **Lys39 HZ3 - ATP O2B |
|  |  |  |
| **Lys39 HZ1 - ATP O2G | **Lys39 HZ2 - ATP O2G | **Lys39 HZ3 - ATP O2G |
|  |  |  |
| Lys39 HZ1 - ATP O3A | Lys39 HZ2 - ATP O3A | Lys39 HZ3 - ATP O3A |
| X | X | X |
| Lys39 HZ1 - ATP O3B | Lys39 HZ2 - ATP O3B | Lys39 HZ3 - ATP O3B |
| X | X | X |
| Lys39 HZ1 - ATP O3G | Lys39 HZ2 - ATP O3G | Lys39 HZ3 - ATP O3G |
|  |  |  |

| **R50P: Lys185(4)/Arg54(3)/Gln52(1)/Lys39(3)** | | |
| --- | --- | --- |
| **REPLICA 1** | | |
| **Monomer 1** | | |
| Lys185 HZ1 – ATP O1A | Lys185 HZ2 – ATP O1A | Lys185 HZ3 – ATP O1A |
|  |  |  |
| Lys185 HZ1 – ATP O1B | Lys185 HZ2 – ATP O1B | Lys185 HZ3 – ATP O1B |
| X | X | X |
| Lys185 HZ1 – ATP O1G | Lys185 HZ2 – ATP O1G | Lys185 HZ3 – ATP O1G |
|  |  |  |
| Lys185 HZ1 – ATP O2A | Lys185 HZ2 – ATP O2A | Lys185 HZ3 – ATP O2A |
| X | X | X |
| Lys185 HZ1 – ATP O2B | Lys185 HZ2 – ATP O2B | Lys185 HZ3 – ATP O2B |
|  |  |  |
| Lys185 HZ1 – ATP O2G | Lys185 HZ2 – ATP O2G | Lys185 HZ3 – ATP O2G |
|  |  |  |
| Lys185 HZ1 – ATP O3A | Lys185 HZ2 – ATP O3A | Lys185 HZ3 – ATP O3A |
| X | X | X |
| Lys185 HZ1 – ATP O3B | Lys185 HZ2 – ATP O3B | Lys185 HZ3 – ATP O3B |
|  |  |  |
| Lys185 HZ1 – ATP O3G | Lys185 HZ2 – ATP O3G | Lys185 HZ3 – ATP O3G |
|  |  |  |
| Arg54 HH11 – ATP O1A | Arg54 HH12 – ATP O1A |  |
| X | X |  |
| Arg54 HH11 – ATP O1B | Arg54 HH12 – ATP O1B |  |
| X | X |  |
| Arg54 HH11 – ATP O1G | Arg54 HH12 – ATP O1G |  |
| X |  |  |
| Arg54 HH21 – ATP O1A | Arg54 HH22 – ATP O1A |  |
| X | X |  |
| Arg54 HH21 – ATP O1B | Arg54 HH22 – ATP O1B |  |
| X | X |  |
| Arg54 HH21 – ATP O1G | Arg54 HH22 – ATP O1G |  |
| X |  |  |
| Arg54 HH11 – ATP O2A | Arg54 HH12 – ATP O2A |  |
| X | X |  |
| Arg54 HH11 – ATP O2B | Arg54 HH12 – ATP O2B |  |
| X | X |  |
| Arg54 HH11 – ATP O2G | Arg54 HH12 – ATP O2G |  |
| X |  |  |
| Arg54 HH21 – ATP O2A | Arg54 HH22 – ATP O2A |  |
| X | X |  |
| Arg54 HH21 – ATP O2B | Arg54 HH22 – ATP O2B |  |
| X | X |  |
| Arg54 HH21 – ATP O2G | Arg54 HH22 – ATP O2G |  |
| X |  |  |
| Arg54 HH11 – ATP O3A | Arg54 HH12 – ATP O3A |  |
| X | X |  |
| Arg54 HH11 – ATP O3B | Arg54 HH12 – ATP O3B |  |
| X | X |  |
| Arg54 HH11 – ATP O3G | Arg54 HH12 – ATP O3G |  |
| X |  |  |
| Arg54 HH21 – ATP O3A | Arg54 HH22 – ATP O3A |  |
| X |  |  |
| Arg54 HH21 – ATP O3B | Arg54 HH22 – ATP O3B |  |
| X |  |  |
| Arg54 HH21 – ATP O3G | Arg54 HH22 – ATP O3G |  |
| X |  |  |
| Gln52 HE21 - ATP O1A | Gln52 HE22 - ATP O1A |  |
| X | X |  |
| **Gln52 HE21 - ATP O1B | **Gln52 HE22 - ATP O1B |  |
|  |  |  |
| Gln52 HE21 - ATP O1G | Gln52 HE22 - ATP O1G |  |
| X | X |  |
| **Gln52 HE21 - ATP O2A | **Gln52 HE22 - ATP O2A |  |
|  |  |  |
| Gln52 HE21 - ATP O2B | Gln52 HE22 - ATP O2B |  |
| X | X |  |
| Gln52 HE21 - ATP O2G | Gln52 HE22 - ATP O2G |  |
| X | X |  |
| Gln52 HE21 - ATP O3A | Gln52 HE22 - ATP O3A |  |
| X | X |  |
| Gln52 HE21 - ATP O3B | Gln52 HE22 - ATP O3B |  |
| X | X |  |
| Gln52 HE21 - ATP O3G | Gln52 HE22 - ATP O3G |  |
| X | X |  |
| Lys39 HZ1 - ATP O1A | Lys39 HZ2 - ATP O1A | Lys39 HZ3 - ATP O1A |
| X | X | X |
| Lys39 HZ1 - ATP O1B | Lys39 HZ2 - ATP O1B | Lys39 HZ3 - ATP O1B |
| X | X | X |
| Lys39 HZ1 - ATP O1G | Lys39 HZ2 - ATP O1G | Lys39 HZ3 - ATP O1G |
|  |  |  |
| Lys39 HZ1 - ATP O2A | Lys39 HZ2 - ATP O2A | Lys39 HZ3 - ATP O2A |
| X | X | X |
| Lys39 HZ1 - ATP O2B | Lys39 HZ2 - ATP O2B | Lys39 HZ3 - ATP O2B |
|  |  |  |
| Lys39 HZ1 - ATP O2G | Lys39 HZ2 - ATP O2G | Lys39 HZ3 - ATP O2G |
|  |  |  |
| Lys39 HZ1 - ATP O3A | Lys39 HZ2 - ATP O3A | Lys39 HZ3 - ATP O3A |
| X | X | X |
| Lys39 HZ1 - ATP O3B | Lys39 HZ2 - ATP O3B | Lys39 HZ3 - ATP O3B |
|  |  |  |
| Lys39 HZ1 - ATP O3G | Lys39 HZ2 - ATP O3G | Lys39 HZ3 - ATP O3G |
|  |  |  |
| **Monomer 2** | | |
| Lys185 HZ1 – ATP O1A | Lys185 HZ2 – ATP O1A | Lys185 HZ3 – ATP O1A |
|  |  |  |
| Lys185 HZ1 – ATP O1B | Lys185 HZ2 – ATP O1B | Lys185 HZ3 – ATP O1B |
| X | X | X |
| Lys185 HZ1 – ATP O1G | Lys185 HZ2 – ATP O1G | Lys185 HZ3 – ATP O1G |
|  |  |  |
| Lys185 HZ1 – ATP O2A | Lys185 HZ2 – ATP O2A | Lys185 HZ3 – ATP O2A |
| X | X | X |
| Lys185 HZ1 – ATP O2B | Lys185 HZ2 – ATP O2B | Lys185 HZ3 – ATP O2B |
|  |  |  |
| Lys185 HZ1 – ATP O2G | Lys185 HZ2 – ATP O2G | Lys185 HZ3 – ATP O2G |
|  |  |  |
| Lys185 HZ1 – ATP O3A | Lys185 HZ2 – ATP O3A | Lys185 HZ3 – ATP O3A |
|  |  |  |
| Lys185 HZ1 – ATP O3B | Lys185 HZ2 – ATP O3B | Lys185 HZ3 – ATP O3B |
|  |  |  |
| **Lys185 HZ1 – ATP O3G | **Lys185 HZ2 – ATP O3G | **Lys185 HZ3 – ATP O3G |
|  |  |  |
| Arg54 HH11 – ATP O1A | Arg54 HH12 – ATP O1A |  |
| X | X |  |
| Arg54 HH11 – ATP O1B | Arg54 HH12 – ATP O1B |  |
| X | X |  |
| Arg54 HH11 – ATP O1G | Arg54 HH12 – ATP O1G |  |
|  |  |  |
| Arg54 HH21 – ATP O1A | Arg54 HH22 – ATP O1A |  |
| X | X |  |
| Arg54 HH21 – ATP O1B | Arg54 HH22 – ATP O1B |  |
| X | X |  |
| Arg54 HH21 – ATP O1G | Arg54 HH22 – ATP O1G |  |
| X |  |  |
| Arg54 HH11 – ATP O2A | Arg54 HH12 – ATP O2A |  |
| X | X |  |
| Arg54 HH11 – ATP O2B | Arg54 HH12 – ATP O2B |  |
| X | X |  |
| Arg54 HH11 – ATP O2G | Arg54 HH12 – ATP O2G |  |
| X |  |  |
| Arg54 HH21 – ATP O2A | Arg54 HH22 – ATP O2A |  |
| X | X |  |
| Arg54 HH21 – ATP O2B | Arg54 HH22 – ATP O2B |  |
| X | X |  |
| Arg54 HH21 – ATP O2G | Arg54 HH22 – ATP O2G |  |
| X | X |  |
| Arg54 HH11 – ATP O3A | Arg54 HH12 – ATP O3A |  |
| X | X |  |
| Arg54 HH11 – ATP O3B | Arg54 HH12 – ATP O3B |  |
| X |  |  |
| Arg54 HH11 – ATP O3G | Arg54 HH12 – ATP O3G |  |
| X |  |  |
| Arg54 HH21 – ATP O3A | Arg54 HH22 – ATP O3A |  |
| X | X |  |
| Arg54 HH21 – ATP O3B | Arg54 HH22 – ATP O3B |  |
| X |  |  |
| Arg54 HH21 – ATP O3G | Arg54 HH22 – ATP O3G |  |
| X |  |  |
| Lys39 HZ1 - ATP O1A | Lys39 HZ2 - ATP O1A | Lys39 HZ3 - ATP O1A |
| X | X | X |
| Lys39 HZ1 - ATP O1B | Lys39 HZ2 - ATP O1B | Lys39 HZ3 - ATP O1B |
| X | X | X |
| Lys39 HZ1 - ATP O1G | Lys39 HZ2 - ATP O1G | Lys39 HZ3 - ATP O1G |
|  |  |  |
| Lys39 HZ1 - ATP O2A | Lys39 HZ2 - ATP O2A | Lys39 HZ3 - ATP O2A |
| X | X | X |
| Lys39 HZ1 - ATP O2B | Lys39 HZ2 - ATP O2B | Lys39 HZ3 - ATP O2B |
| X | X | X |
| Lys39 HZ1 - ATP O2G | Lys39 HZ2 - ATP O2G | Lys39 HZ3 - ATP O2G |
|  |  |  |
| Lys39 HZ1 - ATP O3A | Lys39 HZ2 - ATP O3A | Lys39 HZ3 - ATP O3A |
| X | X | X |
| Lys39 HZ1 - ATP O3B | Lys39 HZ2 - ATP O3B | Lys39 HZ3 - ATP O3B |
| X | X | X |
| Lys39 HZ1 - ATP O3G | Lys39 HZ2 - ATP O3G | Lys39 HZ3 - ATP O3G |
|  |  |  |
| **Monomer 3** | | |
| Lys185 HZ1 – ATP O1A | Lys185 HZ2 – ATP O1A | Lys185 HZ3 – ATP O1A |
|  |  |  |
| Lys185 HZ1 – ATP O1B | Lys185 HZ2 – ATP O1B | Lys185 HZ3 – ATP O1B |
| X | X | X |
| **Lys185 HZ1 – ATP O1G | **Lys185 HZ2 – ATP O1G | **Lys185 HZ3 – ATP O1G |
|  |  |  |
| Lys185 HZ1 – ATP O2A | Lys185 HZ2 – ATP O2A | Lys185 HZ3 – ATP O2A |
| X | X | X |
| Lys185 HZ1 – ATP O2B | Lys185 HZ2 – ATP O2B | Lys185 HZ3 – ATP O2B |
|  |  |  |
| **Lys185 HZ1 – ATP O2G | **Lys185 HZ2 – ATP O2G | **Lys185 HZ3 – ATP O2G |
|  |  |  |
| Lys185 HZ1 – ATP O3A | **Lys185 HZ2 – ATP O3A | **Lys185 HZ3 – ATP O3A |
| X |  |  |
| **Lys185 HZ1 – ATP O3B | **Lys185 HZ2 – ATP O3B | **Lys185 HZ3 – ATP O3B |
|  |  |  |
| **Lys185 HZ1 – ATP O3G | **Lys185 HZ2 – ATP O3G | **Lys185 HZ3 – ATP O3G |
|  |  |  |
| **Monomer 4** | | |
| Lys185 HZ1 – ATP O1A | Lys185 HZ2 – ATP O1A | Lys185 HZ3 – ATP O1A |
|  |  |  |
| Lys185 HZ1 – ATP O1B | Lys185 HZ2 – ATP O1B | Lys185 HZ3 – ATP O1B |
|  |  |  |
| **Lys185 HZ1 – ATP O1G | **Lys185 HZ2 – ATP O1G | **Lys185 HZ3 – ATP O1G |
|  |  |  |
| Lys185 HZ1 – ATP O2A | Lys185 HZ2 – ATP O2A | Lys185 HZ3 – ATP O2A |
|  |  |  |
| Lys185 HZ1 – ATP O2B | Lys185 HZ2 – ATP O2B | Lys185 HZ3 – ATP O2B |
|  |  |  |
| **Lys185 HZ1 – ATP O2G | **Lys185 HZ2 – ATP O2G | **Lys185 HZ3 – ATP O2G |
|  |  |  |
| Lys185 HZ1 – ATP O3A | Lys185 HZ2 – ATP O3A | Lys185 HZ3 – ATP O3A |
| X | X | X |
| Lys185 HZ1 – ATP O3B | Lys185 HZ2 – ATP O3B | Lys185 HZ3 – ATP O3B |
|  |  |  |
| **Lys185 HZ1 – ATP O3G | **Lys185 HZ2 – ATP O3G | **Lys185 HZ3 – ATP O3G |
|  |  |  |
| Arg54 HH11 – ATP O1A | Arg54 HH12 – ATP O1A |  |
| X | X |  |
| Arg54 HH11 – ATP O1B | Arg54 HH12 – ATP O1B |  |
| X |  |  |
| Arg54 HH11 – ATP O1G | Arg54 HH12 – ATP O1G |  |
| X |  |  |
| Arg54 HH21 – ATP O1A | Arg54 HH22 – ATP O1A |  |
| X | X |  |
| Arg54 HH21 – ATP O1B | Arg54 HH22 – ATP O1B |  |
| X |  |  |
| Arg54 HH21 – ATP O1G | Arg54 HH22 – ATP O1G |  |
| X |  |  |
| Arg54 HH11 – ATP O2A | Arg54 HH12 – ATP O2A |  |
| X | X |  |
| Arg54 HH11 – ATP O2B | Arg54 HH12 – ATP O2B |  |
| X | X |  |
| Arg54 HH11 – ATP O2G | Arg54 HH12 – ATP O2G |  |
| X |  |  |
| Arg54 HH21 – ATP O2A | Arg54 HH22 – ATP O2A |  |
| X | X |  |
| Arg54 HH21 – ATP O2B | Arg54 HH22 – ATP O2B |  |
| X | X |  |
| Arg54 HH21 – ATP O2G | Arg54 HH22 – ATP O2G |  |
| X |  |  |
| Arg54 HH11 – ATP O3A | Arg54 HH12 – ATP O3A |  |
| X |  |  |
| Arg54 HH11 – ATP O3B | Arg54 HH12 – ATP O3B |  |
| X |  |  |
| Arg54 HH11 – ATP O3G | Arg54 HH12 – ATP O3G |  |
| X |  |  |
| Arg54 HH21 – ATP O3A | Arg54 HH22 – ATP O3A |  |
| X |  |  |
| Arg54 HH21 – ATP O3B | Arg54 HH22 – ATP O3B |  |
| X |  |  |
| Arg54 HH21 – ATP O3G | Arg54 HH22 – ATP O3G |  |
| X |  |  |
| Lys39 HZ1 - ATP O1A | Lys39 HZ2 - ATP O1A | Lys39 HZ3 - ATP O1A |
| X | X | X |
| Lys39 HZ1 - ATP O1B | Lys39 HZ2 - ATP O1B | Lys39 HZ3 - ATP O1B |
| X | X | X |
| Lys39 HZ1 - ATP O1G | Lys39 HZ2 - ATP O1G | Lys39 HZ3 - ATP O1G |
|  |  |  |
| Lys39 HZ1 - ATP O2A | Lys39 HZ2 - ATP O2A | Lys39 HZ3 - ATP O2A |
| X | X | X |
| Lys39 HZ1 - ATP O2B | Lys39 HZ2 - ATP O2B | Lys39 HZ3 - ATP O2B |
|  |  |  |
| Lys39 HZ1 - ATP O2G | Lys39 HZ2 - ATP O2G | Lys39 HZ3 - ATP O2G |
|  |  |  |
| Lys39 HZ1 - ATP O3A | Lys39 HZ2 - ATP O3A | Lys39 HZ3 - ATP O3A |
| X | X | X |
| Lys39 HZ1 - ATP O3B | Lys39 HZ2 - ATP O3B | Lys39 HZ3 - ATP O3B |
|  |  |  |
| Lys39 HZ1 - ATP O3G | Lys39 HZ2 - ATP O3G | Lys39 HZ3 - ATP O3G |
|  |  |  |

| **R50P: Lys185(4)/Arg54(3)/Gln52(2)/Lys39(3)** | | |
| --- | --- | --- |
| **REPLICA 2** | | |
| **Monomer 1** | | |
| Lys185 HZ1 – ATP O1A | Lys185 HZ2 – ATP O1A | Lys185 HZ3 – ATP O1A |
|  |  |  |
| Lys185 HZ1 – ATP O1B | Lys185 HZ2 – ATP O1B | Lys185 HZ3 – ATP O1B |
| X | X | X |
| Lys185 HZ1 – ATP O1G | Lys185 HZ2 – ATP O1G | Lys185 HZ3 – ATP O1G |
|  |  |  |
| Lys185 HZ1 – ATP O2A | Lys185 HZ2 – ATP O2A | Lys185 HZ3 – ATP O2A |
| X | X | X |
| Lys185 HZ1 – ATP O2B | Lys185 HZ2 – ATP O2B | Lys185 HZ3 – ATP O2B |
|  |  |  |
| Lys185 HZ1 – ATP O2G | Lys185 HZ2 – ATP O2G | Lys185 HZ3 – ATP O2G |
|  |  |  |
| Lys185 HZ1 – ATP O3A | Lys185 HZ2 – ATP O3A | Lys185 HZ3 – ATP O3A |
|  |  |  |
| Lys185 HZ1 – ATP O3B | Lys185 HZ2 – ATP O3B | Lys185 HZ3 – ATP O3B |
|  |  |  |
| Lys185 HZ1 – ATP O3G | Lys185 HZ2 – ATP O3G | Lys185 HZ3 – ATP O3G |
|  |  |  |
| **Monomer 2** | | |
| Lys185 HZ1 – ATP O1A | Lys185 HZ2 – ATP O1A | Lys185 HZ3 – ATP O1A |
|  |  |  |
| Lys185 HZ1 – ATP O1B | Lys185 HZ2 – ATP O1B | Lys185 HZ3 – ATP O1B |
| X | X | X |
| **Lys185 HZ1 – ATP O1G | **Lys185 HZ2 – ATP O1G | **Lys185 HZ3 – ATP O1G |
|  |  |  |
| Lys185 HZ1 – ATP O2A | Lys185 HZ2 – ATP O2A | Lys185 HZ3 – ATP O2A |
| X | X | X |
| Lys185 HZ1 – ATP O2B | Lys185 HZ2 – ATP O2B | Lys185 HZ3 – ATP O2B |
|  |  |  |
| Lys185 HZ1 – ATP O2G | Lys185 HZ2 – ATP O2G | Lys185 HZ3 – ATP O2G |
|  |  |  |
| Lys185 HZ1 – ATP O3A | Lys185 HZ2 – ATP O3A | Lys185 HZ3 – ATP O3A |
|  |  |  |
| Lys185 HZ1 – ATP O3B | Lys185 HZ2 – ATP O3B | Lys185 HZ3 – ATP O3B |
|  |  |  |
| Lys185 HZ1 – ATP O3G | Lys185 HZ2 – ATP O3G | Lys185 HZ3 – ATP O3G |
|  |  |  |
| Arg54 HH11 – ATP O1A | Arg54 HH12 – ATP O1A |  |
| X | X |  |
| Arg54 HH11 – ATP O1B | Arg54 HH12 – ATP O1B |  |
| X |  |  |
| Arg54 HH11 – ATP O1G | Arg54 HH12 – ATP O1G |  |
| X |  |  |
| Arg54 HH21 – ATP O1A | Arg54 HH22 – ATP O1A |  |
| X | X |  |
| Arg54 HH21 – ATP O1B | Arg54 HH22 – ATP O1B |  |
| X |  |  |
| Arg54 HH21 – ATP O1G | Arg54 HH22 – ATP O1G |  |
| X |  |  |
| Arg54 HH11 – ATP O2A | Arg54 HH12 – ATP O2A |  |
| X | X |  |
| Arg54 HH11 – ATP O2B | Arg54 HH12 – ATP O2B |  |
| X | X |  |
| Arg54 HH11 – ATP O2G | **Arg54 HH12 – ATP O2G |  |
| X |  |  |
| Arg54 HH21 – ATP O2A | Arg54 HH22 – ATP O2A |  |
| X | X |  |
| Arg54 HH21 – ATP O2B | Arg54 HH22 – ATP O2B |  |
| X | X |  |
| Arg54 HH21 – ATP O2G | **Arg54 HH22 – ATP O2G |  |
| X |  |  |
| Arg54 HH11 – ATP O3A | Arg54 HH12 – ATP O3A |  |
| X | X |  |
| Arg54 HH11 – ATP O3B | Arg54 HH12 – ATP O3B |  |
| X |  |  |
| Arg54 HH11 – ATP O3G | Arg54 HH12 – ATP O3G |  |
| X |  |  |
| Arg54 HH21 – ATP O3A | Arg54 HH22 – ATP O3A |  |
| X |  |  |
| Arg54 HH21 – ATP O3B | Arg54 HH22 – ATP O3B |  |
| X |  |  |
| Arg54 HH21 – ATP O3G | Arg54 HH22 – ATP O3G |  |
| X |  |  |
| Lys39 HZ1 - ATP O1A | Lys39 HZ2 - ATP O1A | Lys39 HZ3 - ATP O1A |
| X | X | X |
| Lys39 HZ1 - ATP O1B | Lys39 HZ2 - ATP O1B | Lys39 HZ3 - ATP O1B |
| X | X | X |
| Lys39 HZ1 - ATP O1G | Lys39 HZ2 - ATP O1G | Lys39 HZ3 - ATP O1G |
|  |  |  |
| Lys39 HZ1 - ATP O2A | Lys39 HZ2 - ATP O2A | Lys39 HZ3 - ATP O2A |
| X | X | X |
| Lys39 HZ1 - ATP O2B | Lys39 HZ2 - ATP O2B | Lys39 HZ3 - ATP O2B |
|  |  |  |
| Lys39 HZ1 - ATP O2G | Lys39 HZ2 - ATP O2G | Lys39 HZ3 - ATP O2G |
|  |  |  |
| Lys39 HZ1 - ATP O3A | Lys39 HZ2 - ATP O3A | Lys39 HZ3 - ATP O3A |
| X | X | X |
| Lys39 HZ1 - ATP O3B | Lys39 HZ2 - ATP O3B | Lys39 HZ3 - ATP O3B |
| X | X | X |
| Lys39 HZ1 - ATP O3G | Lys39 HZ2 - ATP O3G | Lys39 HZ3 - ATP O3G |
|  |  |  |
| **Monomer 3** | | |
| Lys185 HZ1 – ATP O1A | Lys185 HZ2 – ATP O1A | Lys185 HZ3 – ATP O1A |
|  |  |  |
| Lys185 HZ1 – ATP O1B | Lys185 HZ2 – ATP O1B | Lys185 HZ3 – ATP O1B |
| X | X | X |
| Lys185 HZ1 – ATP O1G | Lys185 HZ2 – ATP O1G | Lys185 HZ3 – ATP O1G |
|  |  |  |
| Lys185 HZ1 – ATP O2A | Lys185 HZ2 – ATP O2A | Lys185 HZ3 – ATP O2A |
| X | X | X |
| Lys185 HZ1 – ATP O2B | Lys185 HZ2 – ATP O2B | Lys185 HZ3 – ATP O2B |
|  |  |  |
| Lys185 HZ1 – ATP O2G | Lys185 HZ2 – ATP O2G | Lys185 HZ3 – ATP O2G |
|  |  |  |
| Lys185 HZ1 – ATP O3A | Lys185 HZ2 – ATP O3A | Lys185 HZ3 – ATP O3A |
| X | X | X |
| Lys185 HZ1 – ATP O3B | Lys185 HZ2 – ATP O3B | Lys185 HZ3 – ATP O3B |
|  |  |  |
| Lys185 HZ1 – ATP O3G | Lys185 HZ2 – ATP O3G | Lys185 HZ3 – ATP O3G |
|  |  |  |
| Gln52 HE21 - ATP O1A | Gln52 HE22 - ATP O1A |  |
| X | X |  |
| Gln52 HE21 - ATP O1B | Gln52 HE22 - ATP O1B |  |
|  |  |  |
| Gln52 HE21 - ATP O1G | Gln52 HE22 - ATP O1G |  |
| X | X |  |
| Gln52 HE21 - ATP O2A | Gln52 HE22 - ATP O2A |  |
|  |  |  |
| Gln52 HE21 - ATP O2B | Gln52 HE22 - ATP O2B |  |
| X | X |  |
| Gln52 HE21 - ATP O2G | Gln52 HE22 - ATP O2G |  |
| X | X |  |
| Gln52 HE21 - ATP O3A | Gln52 HE22 - ATP O3A |  |
| X |  |  |
| Gln52 HE21 - ATP O3B | Gln52 HE22 - ATP O3B |  |
|  | X |  |
| Gln52 HE21 - ATP O3G | Gln52 HE22 - ATP O3G |  |
|  | X |  |
| Arg54 HH11 – ATP O1A | Arg54 HH12 – ATP O1A |  |
| X | X |  |
| Arg54 HH11 – ATP O1B | Arg54 HH12 – ATP O1B |  |
| X | X |  |
| Arg54 HH11 – ATP O1G | Arg54 HH12 – ATP O1G |  |
| X |  |  |
| Arg54 HH21 – ATP O1A | Arg54 HH22 – ATP O1A |  |
| X | X |  |
| Arg54 HH21 – ATP O1B | Arg54 HH22 – ATP O1B |  |
| X | X |  |
| Arg54 HH21 – ATP O1G | Arg54 HH22 – ATP O1G |  |
| X |  |  |
| Arg54 HH11 – ATP O2A | Arg54 HH12 – ATP O2A |  |
| X | X |  |
| Arg54 HH11 – ATP O2B | Arg54 HH12 – ATP O2B |  |
| X | X |  |
| Arg54 HH11 – ATP O2G | Arg54 HH12 – ATP O2G |  |
| X |  |  |
| Arg54 HH21 – ATP O2A | Arg54 HH22 – ATP O2A |  |
| X | X |  |
| Arg54 HH21 – ATP O2B | Arg54 HH22 – ATP O2B |  |
| X |  |  |
| Arg54 HH21 – ATP O2G | Arg54 HH22 – ATP O2G |  |
| X | X |  |
| Arg54 HH11 – ATP O3A | Arg54 HH12 – ATP O3A |  |
| X |  |  |
| Arg54 HH11 – ATP O3B | Arg54 HH12 – ATP O3B |  |
| X |  |  |
| Arg54 HH11 – ATP O3G | Arg54 HH12 – ATP O3G |  |
| X |  |  |
| Arg54 HH21 – ATP O3A | Arg54 HH22 – ATP O3A |  |
| X | X |  |
| Arg54 HH21 – ATP O3B | Arg54 HH22 – ATP O3B |  |
| X |  |  |
| Arg54 HH21 – ATP O3G | Arg54 HH22 – ATP O3G |  |
| X |  |  |
| Lys39 HZ1 - ATP O1A | Lys39 HZ2 - ATP O1A | Lys39 HZ3 - ATP O1A |
| X | X | X |
| Lys39 HZ1 - ATP O1B | Lys39 HZ2 - ATP O1B | Lys39 HZ3 - ATP O1B |
| X | X | X |
| Lys39 HZ1 - ATP O1G | Lys39 HZ2 - ATP O1G | Lys39 HZ3 - ATP O1G |
|  |  |  |
| Lys39 HZ1 - ATP O2A | Lys39 HZ2 - ATP O2A | Lys39 HZ3 - ATP O2A |
| X | X | X |
| Lys39 HZ1 - ATP O2B | Lys39 HZ2 - ATP O2B | Lys39 HZ3 - ATP O2B |
|  |  |  |
| Lys39 HZ1 - ATP O2G | Lys39 HZ2 - ATP O2G | Lys39 HZ3 - ATP O2G |
|  |  |  |
| Lys39 HZ1 - ATP O3A | Lys39 HZ2 - ATP O3A | Lys39 HZ3 - ATP O3A |
| X | X | X |
| Lys39 HZ1 - ATP O3B | Lys39 HZ2 - ATP O3B | Lys39 HZ3 - ATP O3B |
|  |  |  |
| Lys39 HZ1 - ATP O3G | Lys39 HZ2 - ATP O3G | Lys39 HZ3 - ATP O3G |
|  |  |  |
| **Monomer 4** | | |
| Lys185 HZ1 – ATP O1A | Lys185 HZ2 – ATP O1A | Lys185 HZ3 – ATP O1A |
|  |  |  |
| Lys185 HZ1 – ATP O1B | Lys185 HZ2 – ATP O1B | Lys185 HZ3 – ATP O1B |
|  |  |  |
| Lys185 HZ1 – ATP O1G | Lys185 HZ2 – ATP O1G | Lys185 HZ3 – ATP O1G |
|  |  |  |
| Lys185 HZ1 – ATP O2A | Lys185 HZ2 – ATP O2A | Lys185 HZ3 – ATP O2A |
|  |  |  |
| Lys185 HZ1 – ATP O2B | Lys185 HZ2 – ATP O2B | Lys185 HZ3 – ATP O2B |
|  |  |  |
| Lys185 HZ1 – ATP O2G | **Lys185 HZ2 – ATP O2G | Lys185 HZ3 – ATP O2G |
|  |  |  |
| Lys185 HZ1 – ATP O3A | Lys185 HZ2 – ATP O3A | Lys185 HZ3 – ATP O3A |
|  |  |  |
| Lys185 HZ1 – ATP O3B | Lys185 HZ2 – ATP O3B | Lys185 HZ3 – ATP O3B |
|  |  |  |
| Lys185 HZ1 – ATP O3G | Lys185 HZ2 – ATP O3G | Lys185 HZ3 – ATP O3G |
|  |  |  |
| Gln52 HE21 - ATP O1A | Gln52 HE22 - ATP O1A |  |
| X | X |  |
| Gln52 HE21 - ATP O1B | Gln52 HE22 - ATP O1B |  |
| X |  |  |
| Gln52 HE21 - ATP O1G | Gln52 HE22 - ATP O1G |  |
| X | X |  |
| Gln52 HE21 - ATP O2A | Gln52 HE22 - ATP O2A |  |
| X |  |  |
| Gln52 HE21 - ATP O2B | Gln52 HE22 - ATP O2B |  |
| X | X |  |
| Gln52 HE21 - ATP O2G | Gln52 HE22 - ATP O2G |  |
| X | X |  |
| **Gln52 HE21 - ATP O3A | **Gln52 HE22 - ATP O3A |  |
|  |  |  |
| Gln52 HE21 - ATP O3B | Gln52 HE22 - ATP O3B |  |
| X | X |  |
| Gln52 HE21 - ATP O3G | Gln52 HE22 - ATP O3G |  |
| X | X |  |
| Arg54 HH11 – ATP O1A | Arg54 HH12 – ATP O1A |  |
| X | X |  |
| Arg54 HH11 – ATP O1B | Arg54 HH12 – ATP O1B |  |
| X | X |  |
| Arg54 HH11 – ATP O1G | Arg54 HH12 – ATP O1G |  |
| X |  |  |
| Arg54 HH21 – ATP O1A | Arg54 HH22 – ATP O1A |  |
| X | X |  |
| Arg54 HH21 – ATP O1B | Arg54 HH22 – ATP O1B |  |
| X | X |  |
| Arg54 HH21 – ATP O1G | Arg54 HH22 – ATP O1G |  |
| X |  |  |
| Arg54 HH11 – ATP O2A | Arg54 HH12 – ATP O2A |  |
| X | X |  |
| Arg54 HH11 – ATP O2B | Arg54 HH12 – ATP O2B |  |
| X |  |  |
| Arg54 HH11 – ATP O2G | Arg54 HH12 – ATP O2G |  |
| X |  |  |
| Arg54 HH21 – ATP O2A | Arg54 HH22 – ATP O2A |  |
| X | X |  |
| Arg54 HH21 – ATP O2B | Arg54 HH22 – ATP O2B |  |
| X | X |  |
| Arg54 HH21 – ATP O2G | Arg54 HH22 – ATP O2G |  |
| X |  |  |
| Arg54 HH11 – ATP O3A | Arg54 HH12 – ATP O3A |  |
| X | X |  |
| Arg54 HH11 – ATP O3B | Arg54 HH12 – ATP O3B |  |
| X | X |  |
| Arg54 HH11 – ATP O3G | Arg54 HH12 – ATP O3G |  |
| X |  |  |
| Arg54 HH21 – ATP O3A | Arg54 HH22 – ATP O3A |  |
| X | X |  |
| Arg54 HH21 – ATP O3B | Arg54 HH22 – ATP O3B |  |
| X |  |  |
| Arg54 HH21 – ATP O3G | Arg54 HH22 – ATP O3G |  |
| X |  |  |
| Lys39 HZ1 - ATP O1A | Lys39 HZ2 - ATP O1A | Lys39 HZ3 - ATP O1A |
| X | X | X |
| **Lys39 HZ1 - ATP O1B | **Lys39 HZ2 - ATP O1B | **Lys39 HZ3 - ATP O1B |
|  |  |  |
| Lys39 HZ1 - ATP O1G | Lys39 HZ2 - ATP O1G | Lys39 HZ3 - ATP O1G |
|  |  |  |
| Lys39 HZ1 - ATP O2A | Lys39 HZ2 - ATP O2A | Lys39 HZ3 - ATP O2A |
| X | X | X |
| **Lys39 HZ1 - ATP O2B | **Lys39 HZ2 - ATP O2B | **Lys39 HZ3 - ATP O2B |
|  |  |  |
| Lys39 HZ1 - ATP O2G | Lys39 HZ2 - ATP O2G | Lys39 HZ3 - ATP O2G |
|  |  |  |
| Lys39 HZ1 - ATP O3A | Lys39 HZ2 - ATP O3A | Lys39 HZ3 - ATP O3A |
| X | X | X |
| Lys39 HZ1 - ATP O3B | Lys39 HZ2 - ATP O3B | Lys39 HZ3 - ATP O3B |
|  |  |  |
| Lys39 HZ1 - ATP O3G | Lys39 HZ2 - ATP O3G | Lys39 HZ3 - ATP O3G |
|  |  |  |
